# ROS Stress Resets Circadian Clocks to Coordinate Pro-Survival Signals

**DOI:** 10.1371/journal.pone.0082006

**Published:** 2013-12-02

**Authors:** Teruya Tamaru, Mitsuru Hattori, Yasuharu Ninomiya, Genki Kawamura, Guillaume Varès, Kousuke Honda, Durga Prasad Mishra, Bing Wang, Ivor Benjamin, Paolo Sassone-Corsi, Takeaki Ozawa, Ken Takamatsu

**Affiliations:** 1 Department of Physiology & Advanced Research Center for Medical Science, Toho University School of Medicine, Tokyo, Japan; 2 Department of Chemistry, School of Science, The University of Tokyo, Tokyo, Japan; 3 Research Center for Radiation Protection, National Institute of Radiological Science, Chiba, Japan; 4 Cell Death Research Laboratory, Endocrinology Division, CSIR-Central Drug Research Institute, Lucknow, India; 5 Division of Cardiology, Internal Medicine, School of Medicine, University of Utah, Salt Lake City, Utah, United States of America; 6 Center for Epigenetics and Metabolism, School of Medicine, University of California Irvine, Irvine, California, United States of America; Karlsruhe Institute of Technology, Germany

## Abstract

Dysfunction of circadian clocks exacerbates various diseases, in part likely due to impaired stress resistance. It is unclear how circadian clock system responds toward critical stresses, to evoke life-protective adaptation. We identified a reactive oxygen species (ROS), H_2_O_2_ -responsive circadian pathway in mammals. Near-lethal doses of ROS-induced critical oxidative stress (cOS) at the branch point of life and death resets circadian clocks, synergistically evoking protective responses for cell survival. The cOS-triggered clock resetting and pro-survival responses are mediated by transcription factor, central clock-regulatory BMAL1 and heat shock stress-responsive (HSR) HSF1. Casein kinase II (CK2) –mediated phosphorylation regulates dimerization and function of BMAL1 and HSF1 to control the cOS-evoked responses. The core cOS-responsive transcriptome includes CK2-regulated crosstalk between the circadian, HSR, NF-kappa-B-mediated anti-apoptotic, and Nrf2-mediated anti-oxidant pathways. This novel circadian-adaptive signaling system likely plays fundamental protective roles in various ROS-inducible disorders, diseases, and death.

## Introduction

As an adaptive response to daily environmental changes, the biological clock (circadian system) confers temporal order as a multi-cellular/tissue synchronization state evoked by resetting cues and daily anticipatory rhythmic physiological processes, called circadian rhythms. It is now understood that most cells in the mammalian body contain autonomous circadian clocks [1,2]. Self-sustained circadian oscillations are generated by molecular clocks via the transcriptional/translational feedback loop [3], which produces global clock-controlled gene (CCG) expression [4,5]. The positive limb of this feedback loop is mediated the bHLH-PAS transcription factors BMAL1 and CLOCK/NPAS2, which form heterodimers, bind to E-box elements, and drive CCG expression [6-8]. Among these target genes are period (*Per*) and cryptochrome (*Cry*), whose protein products accumulate in the cytoplasm where they associate with each other and ultimately translocate to the nucleus. Once in the nucleus, PER and CRY inhibit BMAL1/ CLOCK activity, repressing their own transcription, thus forming the negative limb of the circadian oscillator [3,9]. In parallel, a second feedback loop is generated via RORE-binding activators (Rora, Rorb, Rorc) and repressors (Rev-erb-alpha, Rev-erb-beta), whose transcription is driven by BMAL1/CLOCK [10]. The circadian system mediates fundamental cellular functions, including the cell cycle and energy metabolism and various physiological functions, and is related to diseases such as cancer and metabolic disorders [11,12]. Posttranslational modifications [13-15] such as CK2-mediated circadian BMAL1 phosphorylation play a pivotal role in controlling clock [16]. 

Light and temperature are the two most reliable environmental timing cues for the resetting of circadian clocks [17,18]. In the context of evolution, daily changes in certain stressors on ancient earth, especially very strong solar radiation in the daytime, may cause lethal damage and various diseases, forcing the circadian system to evolve as a daily-regulated protective system. Heat and reactive oxygen species (ROS) are probably general and fundamental inducers of biological disorders as they impose heat and oxidative stress (OS). To determine how the circadian system responds to these stresses, we hypothesized that appropriate doses of stress reset circadian clocks to evoke life-protection systems. We previously demonstrated that an optimal dose of heat shock (HS) resets circadian rhythms [19]. The transcription factor heat shock factor 1 (HSF1) binds to heat shock elements (HSE) and orchestrates the heat shock stress-responsive (HSR) molecular machinery to maintain protein homeostasis [20]. UV and other types of radiation in cosmic rays are ROS-generating stressors. ROS such as superoxide anion (O2^−^), hydrogen peroxide (H_2_O_2_), and hydroxyl radical (•OH) are generated by endogenous metabolic by-products and exogenous sources [21]. If ROS production is left unmanaged, cells may experience oxidative stress due to an imbalance in cellular redox state, leading to genomic damage and eventually cell death. ROS also serve as second messengers to control physiological and pathological processes [22]. Recent studies have focused on circadian-regulated energy metabolism, redox state and intracellular ROS in living systems and diseases [23,24]. 

It is unclear how the circadian system responds to ROS stress. To address this question, we sought to identify a novel clock-related adaptive signaling system evoked at the life–death boundary to mediate protection from the stress. We found that near-lethal ROS stress resets circadian clocks to induce a pro-survival program, and the clock-resetting signal probably coordinates pro-survival signals through an elaborate network of stress-resistant pathways.

## Materials and Methods

### Plasmid Construction

A mouse *Per2* promoter-driven destabilized luciferase reporter (Per2-Luc) was developed as described [16]. Destabilized SLR red luciferase (Toyobo, Japan) reporter connected with 3× HSE (HSE-SLR) was previously constructed [19]. The expression vector for NF-kappa-B-binding element-driven Luc [pGL4.32 (luc2P/NF-kappa-B-RE/Hygro); Promega, Japan] and Nrf2-binding ARE-driven Luc (pNRF2/ARE-Luc Reporter Vector; Signosis, USA) were purchased. The expression vector for CMV promoter-driven Myc-tagged mHSF1 was purchased (OriGene, USA), and a mutant (T142A) was generated using a Quick Change site-directed mutagenesis kit (Stratagene, USA). Retroviral vectors pCLNCX and pMX for *mBmal1* promoter-driven Myc-mBMAL1-WT/S90A and Myc-mHSF1-WT/T142A, respectively, were constructed as described [16]. For the split luciferase complementation assay, Emerald Luciferase (ELuc) cDNA was purchased (Toyobo, Japan). Full-length mouse BMAL1 and HSF1 were ligated downstream of the C-terminal (ELucC) and the N-terminal luciferase fragments (ELucN). Each cDNA fragment was PCR-amplified and inserted into pcDNA4/V5-His (B) or pcDNA3.1 (Invitrogen, Japan) at multi-cloning sites. In the LucC-BMAL1 expression vector, 3 repeats of Rev-erb-alpha/ROR binding element (RRE) in *Bmal1* promoter were added. These experiments were performed with the approval of the committee of Toho University (No. 12-52-145).

### Cell Culture, Transfection, and Retroviral Infection

Mouse NIH-3T3 fibroblasts (RIKEN cell bank, Japan), U2OS (kindly donated by Dr. Nishina), Wild, BMAL1^−/−^ (kindly donated by Dr. Bradfield) [7] and HSF1^−/−^ [25] MEFs (mouse embryonic fibroblasts), were cultured as described [13]. To synchronize the circadian rhythm, cells were cultured to confluence at 37°C in DMEM containing 10% fetal bovine serum, and then treated with various doses of H_2_O_2_ as indicated. Cell culture medium was replaced with DMEM containing 10% fetal bovine serum. DNA transfection was performed using Fugene HD (Roche Applied Science, Japan) according to the manufacturer’s protocol. NIH-3T3-Per2-Luc/HSE-SLR cells and U2OS-Per2 Luc cells were established as described [19]. NIH-3T3:Per2L/HSE-SLR were cOS-pulsed and treated with protein kinase inhibitors for CK2 (I; DMAT, II; TBCA, Calbiochem, USA), CK1 (CKI-7, WAKO, Japan), JNK (L-JNKi1, BIOMOL, USA), p38 (SB203580, Calbiochem, USA), MEK (U0126, BIOMOL, USA) and PKA (inhibitor fragment (6-22) amide, TOCRIS, USA) as well as HSF1 inhibitor (KNK437, Calbiochem, USA). The RetroMax expression system (IMGENEX, USA) was used for the rescue experiments to produce retrovirus. Infection was performed as described [19].

### Real-time Bioluminescence, Fluorescence Assay, and Data Processing

Cells were transfected with plasmids and synchronized as indicated in the figure legends. Real-time bioluminescence in whole cultures with 0.2 mM Luciferin (Toyobo, Japan) were monitored using Kronos (ATTO, Japan) in one-color or dual-color mode and acquisition times of 2 min (promoter-Luc assay) or 3 min (split-Luc assay), according to the manufacturer’s protocol. Values were obtained from each sample using the same detectors in the same experiments. The n-value (n = 3, 4, 5 etc.) indicated for each experiment refers to the number of samples analyzed with the same detectors in the same experiments. If the Y-axis indicates “RLU” (Relative Light Units), the relative photo-counting values were normalized by averaging intensity over time. If the Y-axis indicates “deviation from the moving average,” the values were detrended according to the instrument protocol (Kronos; ATTO, Japan). All detrended values were normalized by averaging intensity over time. The data in the graph were further normalized using maximum circadian peak intensities over time. Real-time bioluminescence/fluorescence for single-cell imaging were monitored on an LV200 (Olympus, Japan) according to the manufacturer’s protocol. Values were normalized to maximum peak intensities over time and to average intensity over time. To test the significance of the circadian rhythmicity, period, and acrophase, we performed computerized analysis of detrended and normalized data in “Cosinor” and “Acro” software downloaded from the Circadian Rhythm Laboratory Software home page (http://www.circadian.org/softwar.html). 

### Confocal Imaging

 Confocal imaging was performed as described [16]; active caspase-3/7 was visualized with CellEvent™ Caspase-3/7 Green (Molecular probes, USA) and nuclei were visualized with DAPI (Molecular probes, USA) on an LSM-510 META microscope (Carl Zeiss, Germany).

### Biochemical Analyses

 Immunoprecipitation and immunoblotting were performed as described [13] using anti-BMAL1 [13], phospho-BMAL1-S90 [19], CLOCK (Affinity Bioreagents, USA), HSF1 (Upstate Biotechnology, USA), phospho-HSF1-T142 (Assay Biotechnology, USA), CK2beta (Calbiochem, USA), CK2alpha, and actin (Santa Cruz Biotechnology, USA) primary antibodies, as well as HRP-conjugated anti-rabbit/goat/mouse secondary IgG (Zymed, USA). The n-value indicated in each figure refers to the number of independent experiments.

### FACS Analysis

Annexin V/PI-FACS for H_2_O_2_-treated cells was performed with the FITC Annexin V/Dead Cell Apoptosis Kit with FITC annexin V and PI for Flow Cytometry (Molecular Probes, USA).

### Statistical Analyses

As described previously [19], we used factorial design analysis of τ-tests to analyze data and calculate p-values, as appropriate.

### Microarray Analysis

Total RNA was extracted from NIH-3T3:Per2-Luc fibroblasts at 4 h (to detect genes that are up-regulated early after a surge of Per2-Luc bioluminescence as monitored by Kronos), 20 h, and 32 h (to detect circadian changes) after treatment with 5 mM H_2_O_2_ for 10 min (in controls, without H_2_O_2_ treatment, medium was exchanged to fresh medium), using the Trizol Plus RNA Purification Kit (Ambion, USA). Microarray hybridizations were performed at Hokkaido System Science Co. Ltd. (Sapporo, Japan) according to the manufacturer’s protocol using the workflow for one-color Mouse GE 4x44K v2 Microarray (Agilent Technologies), which harbor 39430 mouse transcripts (probe sets) (protein coding transcripts cover 79.8% of murine whole genome). The microarray slides were scanned and gene expression profiles analyzed at Hokkaido System Science according to the manufacturer’s protocol. Significance of gene modulations between groups was confirmed by using “Significance Analysis of Microarrays” (SAM, two-class paired) (http://www-stat.stanford.edu/~tibs/SAM/). For functional classification of cOS-regulated genes, we used DAVID [26]. cOS-regulated genes were annotated with data from several public genomic sources, and then a functional classification algorithm clustered genes in a limited number of functionally related groups. The fuzzy heuristic-based procedure allowed us to list several functionally relevant clusters, if necessary. Heatmap visualization for gene expression profiles were performed using Heatmap Builder (http://ashleylab.stanford.edu/tools_scripts.html). To present and explore biological pathways, we used PathVisio [27] and WikiPathways [28]. The fuzzy heuristic-based procedure allowed us to modify figures for pathways, if necessary. Microarray data in NCBI's Gene Expression Omnibus database of the GSE47955 series record can be viewed at the following URL: http://www.ncbi.nlm.nih.gov/geo/query/acc.cgi?acc=GSE47955.

## Results

### Critical Oxidative Stress (cOS) at the Branch Point of Life and Death Resets Clocks

**Figure 1:**
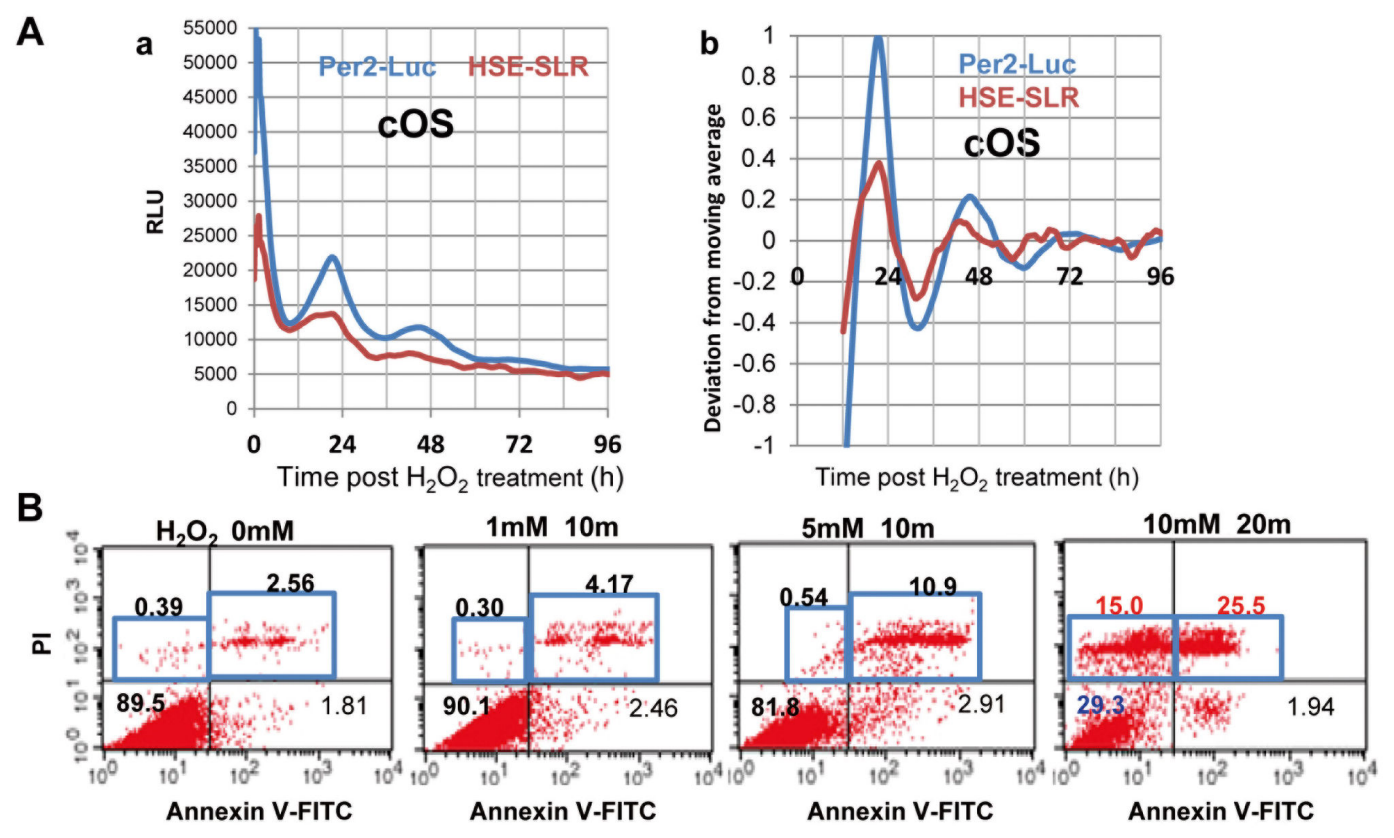
Critical oxidative stress (cOS) at the branch point of life and death resets circadian clocks. (A) NIH-3T3:Per2-Luc /HSE-SLR were cOS-pulsed by treatment with an optimal dose of H_2_O_2_ (5 mM, 10 min) to reset clocks. Circadian Per2-Luc/HSE-SLR profiles were monitored by real-time dual-color bioluminescence assay. Relative (RLU; *a*) and normalized (detrended; deviation from moving average; b) profiles are shown (n = 5). (B) Annexin V/PI-FACS for NIH-3T3 cells after 12 h of various OS doses revealed the critical dose (5 mM, 10 min) for cell survivability. Numerical values indicate the percent of cells belonging to the 4 divided regions.

To test our hypothesis that appropriate doses of stress reset circadian clocks, we investigated whether H_2_O_2_-induced OS resets circadian rhythms. We analyzed the resetting response of NIH-3T3:Per2-Luc/HSE-SLR, NIH-3T3 fibroblasts harboring the *Per2* promoter (BMAL1:CLOCK-transactivated clock gene)-driven luciferase (Luc) and HSE-driven SLR red luciferase (HSE-SLR) reporters [19]. After testing various doses of short-term OS (Figures 1Aab and S1AB), we found that OS (cOS) at the critical dose (millimolar H_2_O_2_ for about 10 min) causes acute Per2-Luc/HSE-SLR elevation followed by overt circadian Per2-Luc oscillation (5 mM H_2_O_2_ for 10 min; Period = 25.3 h, Robustness = 37.4%, Acrophase = 21.72 h, SD in Acrophase = 0.0343). Evident resetting occurred only at >1 mM H_2_O_2_ for 10 min (Figure S1B). Several minutes of OS at millimolar doses of exogenous H_2_O_2_ or other ROS eventually induced cell death including apoptosis; in some cases, these OS act as pivotal intracellular second messengers, activating cellular growth and protective systems as well as pathological conditions such as ischemia-reperfusion injury [28-32]. The temporal profiles of the acute Per2-Luc/HSE-SLR surge (Figure S2A) and circadian Per2-Luc (Figure S2B) (5 mM H_2_O_2_ for 10 min; Period = 26.0 h, Robustness = 57.5%, Acrophase = 35.84 h, SD in Acrophase = 0.0872) demonstrate resetting by OS at similar optimal doses in U2OS:Per2-Luc/HSE-SLR, human osteosarcoma U2OS cells harboring Per2-Luc and HSE-SLR. To verify synchronization by cOS at the single-cell level, temporal Per2-Luc in each U2OS:Per2-Luc was monitored by time-lapse bioluminescence imaging. According to a previous report [2], a circadian rhythm did not emerge in whole cell culture, because it is more likely that each cell has an endogenous rhythm with differential phases under desynchronizing conditions. Appropriate stimulation reveals the overt circadian rhythm in whole cultures by synchronizing the phases of individual cells [1,2]. Consistent with the previous study [2], U2OS:Per2-Luc exhibited no evident synchronous single cellular circadian rhythms for 2 days before the cOS-treatment (Movie S1). After the cOS-pulse, temporal Per2-Luc profiles of single cells exhibited an acute surge and phase synchronization for each circadian rhythm (Figure S2C and Movie S2).

After 1 week of treatment, viable cell counts drastically decreased at doses greater than cOS (Figures S1C and S2D). Flow cytometry with an Annexin V/propidium iodide (PI) assay (Annexin V/PI-FACS) verified that apoptosis and necrosis were remarkably increased, and survivability was drastically reduced by 10 mM H_2_O_2_, in comparison to cOS (1-5mM H_2_O_2_) (Figures 1B). Thus, the critical dose of ROS at the branch point of cellular survival and drastic apoptosis matches the cOS required to reset circadian rhythms. Based on these findings, we believe that we have found a ROS (H_2_O_2_) -dependent circadian control in mammals.

### Circadian/HSR System Regulates Clock Resetting and Cell Survival

**Figure 2:**
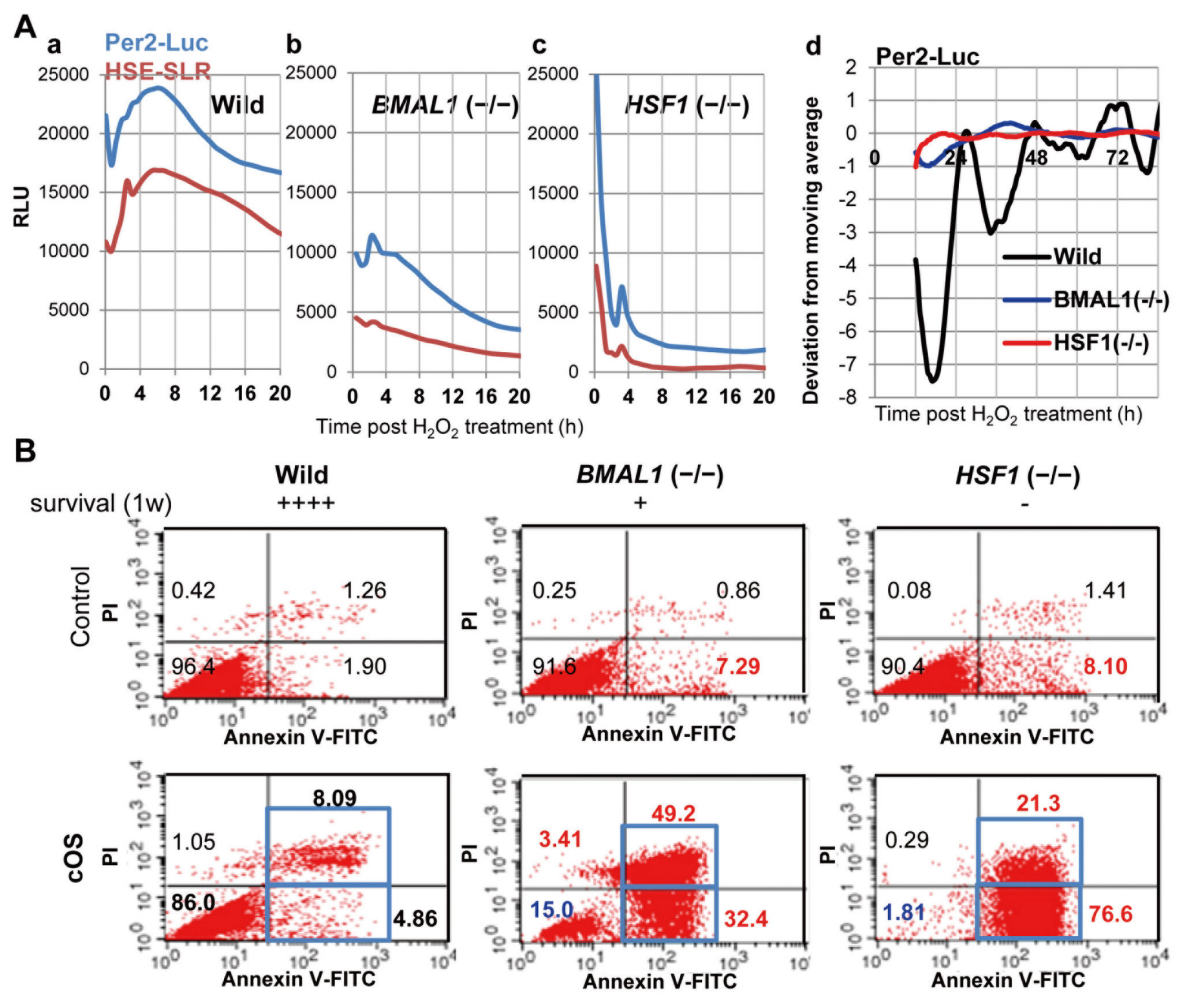
Circadian/HSR systems are indispensable for cOS-evoked responses. (A) HSF1 or BMAL1 deficiency abolishes cOS-synchronized circadian Per2 rhythms and HSE-driven acute surge. Wild-type (Wild) (a,d), BMAL1^−/−^ (b,d), and HSF1^−/−^ (c,d) MEFs transfected with the expression vector for Per2-Luc and HSE-SLR were OS-pulsed. Acute (a-c) and circadian (d) profiles were monitored by real-time bioluminescence assay. Relative (RLU) or normalized (deviation from moving average) profiles are shown (n = 4). (B) Each relative cell survival score 1 week after H_2_O_2_ treatment is shown. The score ++++ indicates 90–100% viable (negative control level), + indicates 25–50% viable, − indicates less than 25% viable (in this case, less than 5% viable). Annexin V/PI-FACS at 12 h post cOS-pulse reveals drastic apoptosis of BMAL1^−/−^ and HSF1^−/−^ MEFs in contrast to WT.

To investigate the role of the circadian/HSR system, we examined the effects of BMAL1/HSF1 deficiency on Per2-Luc rhythms following the cOS pulse, because we hypothesized that the circadian/HSR transcription factors mediate cOS-resetting as they may be involved in HS-resetting [19]. In wild-type (WT) MEFs, we observed an overt circadian Per2-Luc rhythm (Period = 22.4 h, Robustness = 24.8%, Acrophase = 27.0 h, SD in Acrophase = 0.105) preceded by a Per2-Luc/HSE-SLR surge (Figure 2Aad). In contrast, neither an obvious circadian Per2-Luc rhythm nor significant Per2-Luc/HSE-SLR surge was observed in BMAL1^−/−^ and HSF1^−/−^ MEFs (Figures 2Abcd). Importantly, no significant HSE-SLR surge caused by BMAL1 deficiency suggests pivotal involvement of BMAL1 in evoking HSR. Apoptosis and necrosis significantly increased, but survivability decreased in BMAL1^−/−^ and HSF1^−/−^ in comparison to WT (Figure 2B), showing enhanced ROS sensitivity in BMAL1^−/−^ and HSF1^−/−^ cells. Given the anti-apoptotic roles of HSF1 [33-35] and involvement of BMAL1 and HSF1 in anti-oxidant responses [35,36], the cOS-evoked responses probably contribute to cell survival. Our findings demonstrate that HSF1 are indispensable for resetting circadian clocks and survival after cOS pulse. However, BMAL1 is essential for generating the circadian rhythm, and thus it is quite difficult to identify the cause of the disappearance of circadian synchronicity in BMAL1^−/−^ cells.

### CK2 Controls Resetting/Pro-survival Responses via Regulating Circadian-HSR Crosstalk

To characterize the intracellular signaling pathways that mediate resetting and cell survival after cOS-pulse, we screened several candidate signal-transducing protein kinase inhibitors. To limit the sphere of treatment within the synchronization process, the following reversible inhibitors were added 1 h before the cOS-pulse, during the cOS-pulse, and 1 h after the cOS-pulse: NIH-3T3:Per2-Luc/HSE-SLR treated with inhibitors for CK1 (circadian-regulating kinase) [37], JNK [38], p38 (stress-responsive kinases) [39], MEK (ERK-pathway) [39], and PKA (cAMP-pathway) [40] exhibited circadian Per2-Luc rhythms, preceded by a Per2-Luc/HSE-SLR surge after a cOS-pulse similar to the vehicle (Figure S3AB). In contrast, NIH-3T3:Per2-Luc/HSE-SLR treated with CK2 inhibitors (I; DMAT, II; TBCA) and HSF1-mediated transcription inhibitors exhibited a dramatically dampened Per2-Luc rhythm, preceded by a significantly reduced Per2-Luc/HSE-SLR surge. One week after the cOS-pulse, survival was significantly lower in cells treated only with CK2 and HSF1 inhibitors (Figure S3C). Consistently, previous studies have also demonstrated the survival and anti-apoptotic roles of CK2 [41,42]. These data strongly suggest that CK2, as well as HSF1, is pivotal for resetting clocks and cell survival after cOS-pulse.

**Figure 3:**
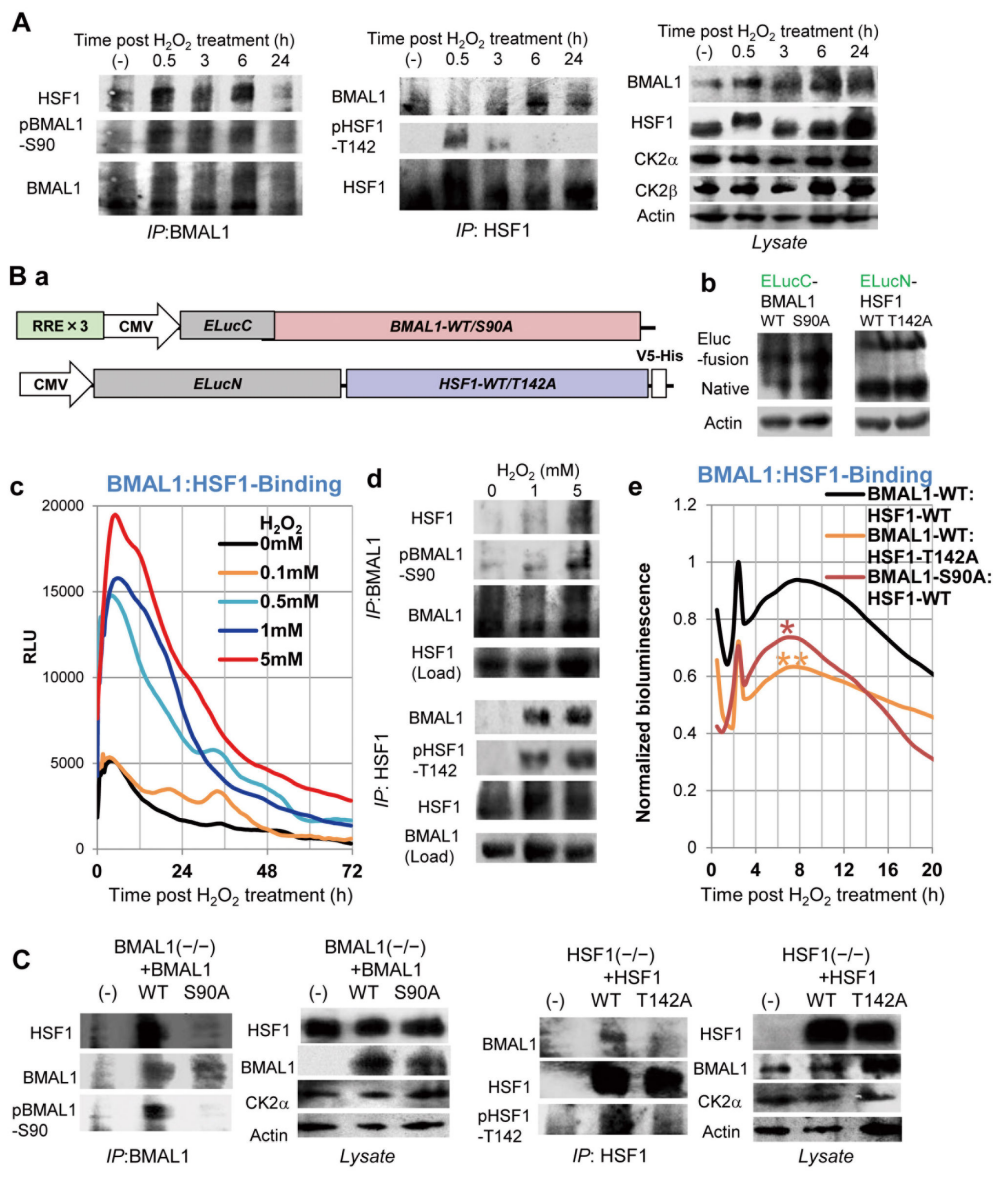
CK2-mediated phosphorylation regulates BMAL1-HSF1 binding. (A) WT MEFs were cOS-pulsed. At the indicated time, BMAL1- and HSF-IP and lysates were analyzed by immunoblotting for BMAL1, phospho-BMAL1-S90 (pBMAL1-S90), HSF1, phospho-HSF1-T142 (pHSF1-T142), CK2alpha, CK2beta, and actin. Representative images are shown (n = 3). (B) CK2 regulates BMAL1-HSF1 binding in living cells. U2OS cells transiently expressing ELucN-HSF1 (WT or T142A lacking a CK2-phospholylation site) and ELucC-BMAL1 (WT or S90A lacking a CK2-phospholylation site) (a; construction map, b; immunoblot detection of recombinant and native proteins) were analyzed by real-time split luciferase complementation assay to detect binding between BMAL1 and HSF1. Relative bioluminescence profiles (n = 4) reveal H_2_O_2_ (treated for 10 min) dose-dependent BMAL1-HSF1 binding (c). BMAL1- and HSF-IP and lysates of U2OS cells after H_2_O_2_ (0, 1, 5 mM) treatment (0.5 h for HSF1-IP or, 4 h for BMAL1-IP) were analyzed by immunoblotting for BMAL1, pBMAL1-S90, HSF1, and pHSF1-T142. Representative images are shown (n = 3) (d). Normalized profiles (n = 4) show a significant difference between WT and the mutants: ⋆ (P<0.05); ⋆⋆ (P< 0.01); at 8 h post cOS-pulse (e). (C) BMAL1^−/−^ MEFs harboring BMAL1-WT or BMAL1-S90A and HSF1^−/−^ MEFs harboring HSF1-WT or HSF1-T142A were cOS-pulsed. At 3 h (30 min for P-HSF1-T142 and P-BMAL1-S90 detection), HSF- and BMAL1-IP and lysates were analyzed by immunoblotting. Representative images are shown (n = 3).

We previously demonstrated acute HSF1-BMAL1 interactions after HS-pulse, suggesting a pivotal role of circadian-HSR crosstalk during HS-pulse -evoked resetting [19]. CK2-mediated BMAL1-Ser90 phosphorylation is indispensable for BMAL1:CLOCK nuclear accumulation and subsequent circadian gene transactivation [16]. CK2-mediated HSF1-Thr142 phosphorylation is important for HSF1 binding to HSEs and subsequent transcription activation [43]. To test the notion that the BMAL1-HSF1 interaction mediates resetting after the cOS-pulse and that the associated response is directly regulated by CK2-mediated phosphorylation, we examined the BMAL1-HSF1 association and their phosphorylation by CK2 after cOS-pulse. First, we performed co-immunoprecipitation and immunoblot analyses. BMAL1-immunoprecipitate (IP) from WT MEFs 0.5–6 h after cOS-pulse, consistent with temporal Per2 elevation, contained higher levels of HSF1 than without cOS-pulse (Figure 3A). HSF1-IP after cOS-pulse contained higher levels of BMAL1 than without cOS-pulse. As in the previous case, after the HS-pulse [19], HSF1-BMAL1 interactions were more frequent in BMAL1-co-IP than in HSF1-co-IP, indicating that HSF1 comprises a greater portion of BMAL1-co-IP than BMAL1 does in HSF1-co-IP. Importantly, CK2-mediated BMAL1-S90/HSF1-T142 phosphorylation increased after cOS-pulse (Figure 3A). The timing of the increase in BMAL1-S90 phosphorylation (at 0.5–6 h post cOS pulse) is consistent with the BMAL1-HSF1 co-IP, preceded by elevated HSF1-T142 phosphorylation (at 0.5 h). These results suggest involvement of CK2-mediated BMAL1/HSF1 phosphorylation in regulating BMAL1-HSF1 interaction. Recruitment of HSF1 to the BMAL1:CLOCK complex via these mechanisms may mediate cOS-resetting. 

To address the question of whether BMAL1 interacts directly with HSF1 and whether CK2-mediated phosphorylation regulates this interaction, we performed a split luciferase complementation assay [44], in which real-time bioluminescence can only be detected when N- (ELucN) and C- (ELucC) terminal luciferase fragments complement each other to generate luciferase activity via formation of BMAL1-HSF1 complex. This method rules out the possibility of nonspecific associations that may occur in IP. For this, we constructed expression vectors for ELucN-HSF1-WT/T142A and ELucC-BMAL1-WT/S90A (wild/CK2 phosphorylation-deficient mutant) (Figure 3Ba). After transfection of these vectors into U2OS cells, ELucN-HSF11 (~100 kDa) and ELucC-BMAL1 (~90 kDa) proteins could be detected by immunoblotting at similar levels as native proteins (Figure 3Bb). We monitored the surge of BMAL1–HSF1 (WT/WT) binding in real-time after the cOS pulse in an H_2_O_2_-dose dependent manner, demonstrating BMAL1-HSF1 complex formation in the cells (Figure 3Bc). This timing is consistent with the BMAL1-HSF1 co-IP pattern (Figure 3A). Additionally, BMAL1-S90 phosphorylation, HSF1-T142 phosphorylation and BMAL1-HSF1 binding occurred in an H_2_O_2_-dose dependent manner (Figure 3Bd). Next, to clarify the role of CK2, we examined the effects of deficiencies in CK2-mediated BMAL1/HSF1 phosphorylation on BMAL1–HSF1 binding. Bioluminescence reflecting binding activity was significantly reduced for BMAL1–WT:HSF1-T142A and BMAL1-S90A:HSF1-WT in comparison to BMAL1-WT:HSF1-WT (Figure 3Be), indicating that CK2-mediated BMAL1/HSF1 phosphorylation is required for BMAL1-HSF1 dimerization after the cOS-pulse in living cells. 

**Figure 4:**
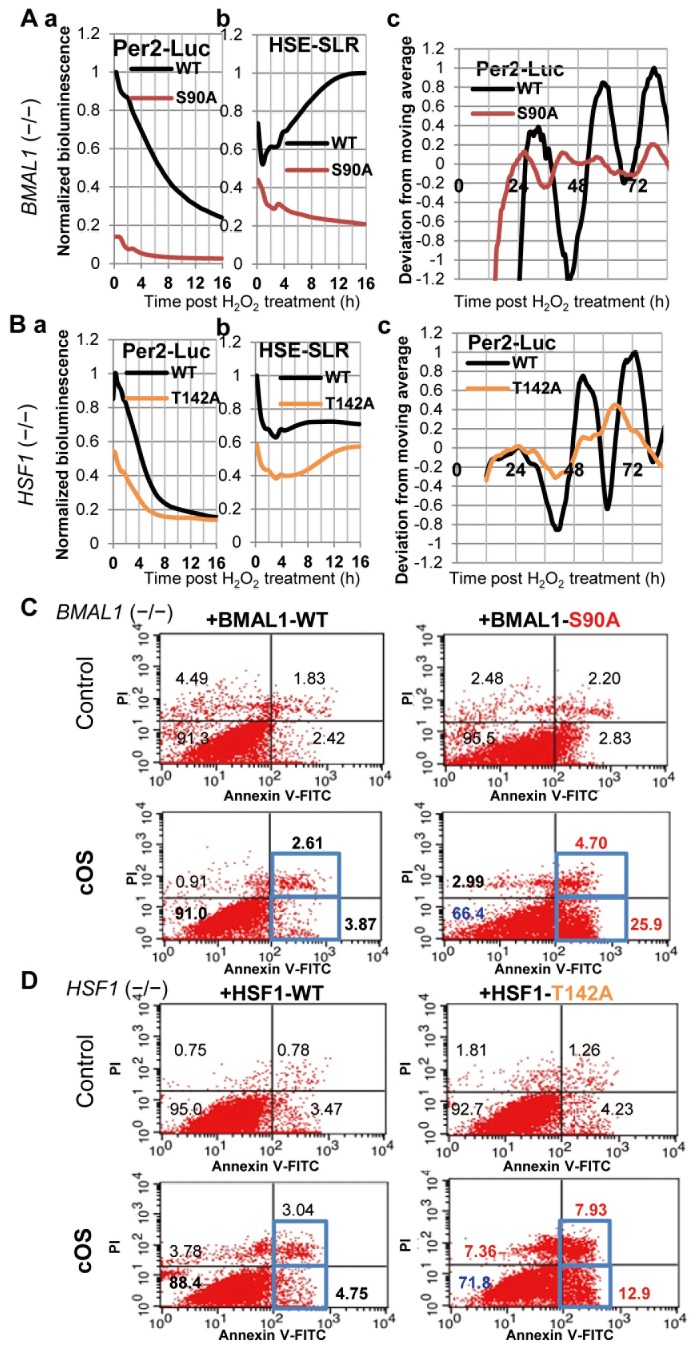
CK2-mediated BMAL1/HSF1 phosphorylation controls cOS-evoked responses. BMAL1^−/−^ MEFs harboring BMAL1-WT or BMAL1-S90A (A,C) and HSF1^−/−^ MEFs harboring HSF1-WT or HSF1-T142A (B,D) were cOS-pulsed. (A,B) Acute (a,b) and circadian (c) Per2-Luc/HSE-SLR profiles are shown as determined by real-time bioluminescence assay (n = 4). (C,D) Annexin V/PI-FACS at 8 h post cOS-pulse reveals reduced survival of BMAL1-S90A and HSF1-T142A cells.

To elucidate the regulatory role of CK2-mediated BMAL1/HSF1 phosphorylation in cOS-resetting, we analyzed Per2-Luc/HSE-SLR profiles in MEFs harboring mutants lacking CK2-mediated phosphorylation (BMAL1-S90A and HSF1-T142A). We further established a pivotal role of CK2 in regulating BMAL1-HSF1 binding (Figure 3C). In MEFs harboring BMAL1-WT and HSF1-WT, circadian Per2-Luc rhythms (BMAL1-WT; Period = 26.0 h, Robustness = 27.2%, Acrophase = 30.44 h, SD in Acrophase = 0.101, HSF1-WT; Period = 22.0 h, Robustness = 42.8%, Acrophase = 23.11 h, SD in Acrophase = 0.121) preceded by a Per2-Luc/HSE-SLR surge after cOS-pulse were restored (Figure 4AB). MEFs harboring BMAL1-S90A exhibited no circadian Per2-Luc rhythm, consistent with previous findings [16], preceded by a much lower Per2-Luc/HSE-SLR surge (Figure 4A), demonstrating that BMAL1-S90 phosphorylation is indispensable for cOS-resetting. MEFs harboring HSF1-T142A exhibited no circadian Per2-Luc rhythm preceded by a lower Per2-Luc/HSE-SLR surge (Figure 4B), indicating a pivotal role of HSF1-T142 phosphorylation during cOS-resetting. Notably, an HSE-SLR surge was restored in MEFs harboring BMAL1-WT, but significantly impaired in MEFs harboring BMAL1-S90A (Figure 4Ab), demonstrating that BMAL1-S90 phosphorylation up-regulates the HSR after cOS-pulse. Apoptosis and necrosis significantly increased, but survivability decreased in BMAL1-S90A and HSF1-T142A, in comparison to WT (Figure 4CD). Our results demonstrate that CK2-mediated BMAL1/HSF1 phosphorylation regulates cOS-evoked resetting and cell survival, perhaps through independent and/or synergistic phosphorylation of BMAL1/HSF, and subsequent circadian-HSR crosstalk.

### cOS-responsive Circadian Transcriptome

**Figure 5:**
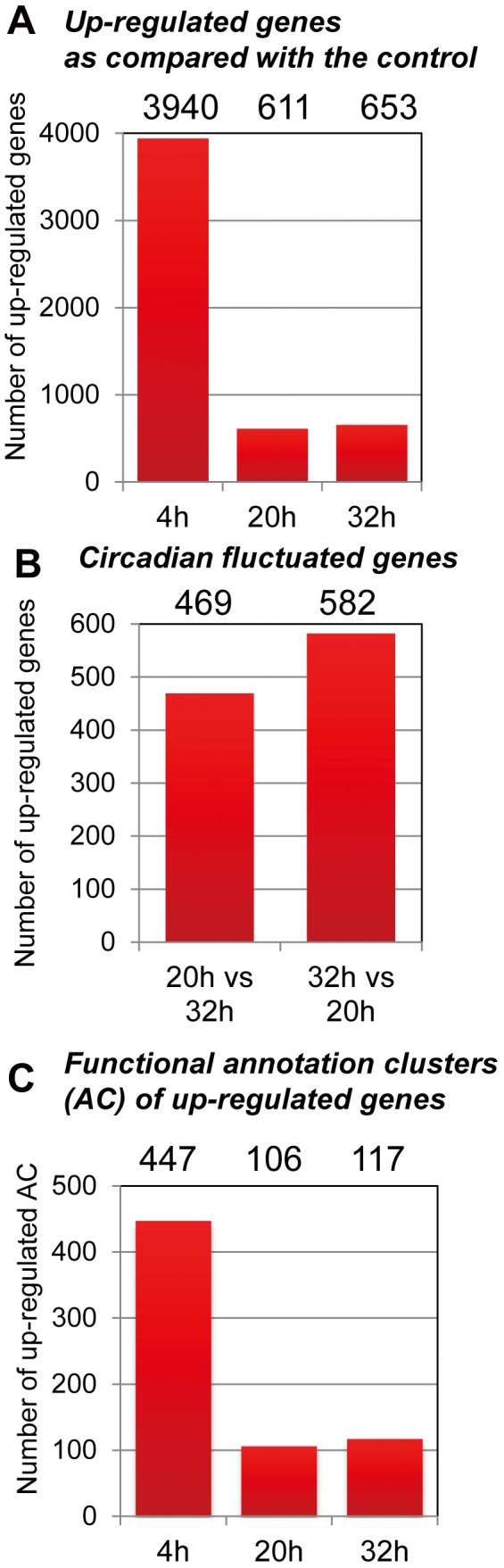
Microarray analysis of gene regulation by cOS-pulse. Microarray analysis using workflow for one-color Mouse GE 4x44K v2 (Agilent Technologies; harboring 39430 mouse transcripts) was performed to identify up-regulated genes during the early stage (4h post cOS-pulse) immediate surge of Per2-Luc and late stage (20h and 32h), as compared vs. the control (only medium-change). (A) The graph shows the total number of the (≧ 2-fold) up-regulated genes at the each time point. (B) The graph shows the total number of the circadian circadian-fluctuated (≧ 2-fold) genes at the each time point. (C) The graph shows the total number of the up-regulated annotation clusters (ACs) at the each time point.

**Figure 6:**
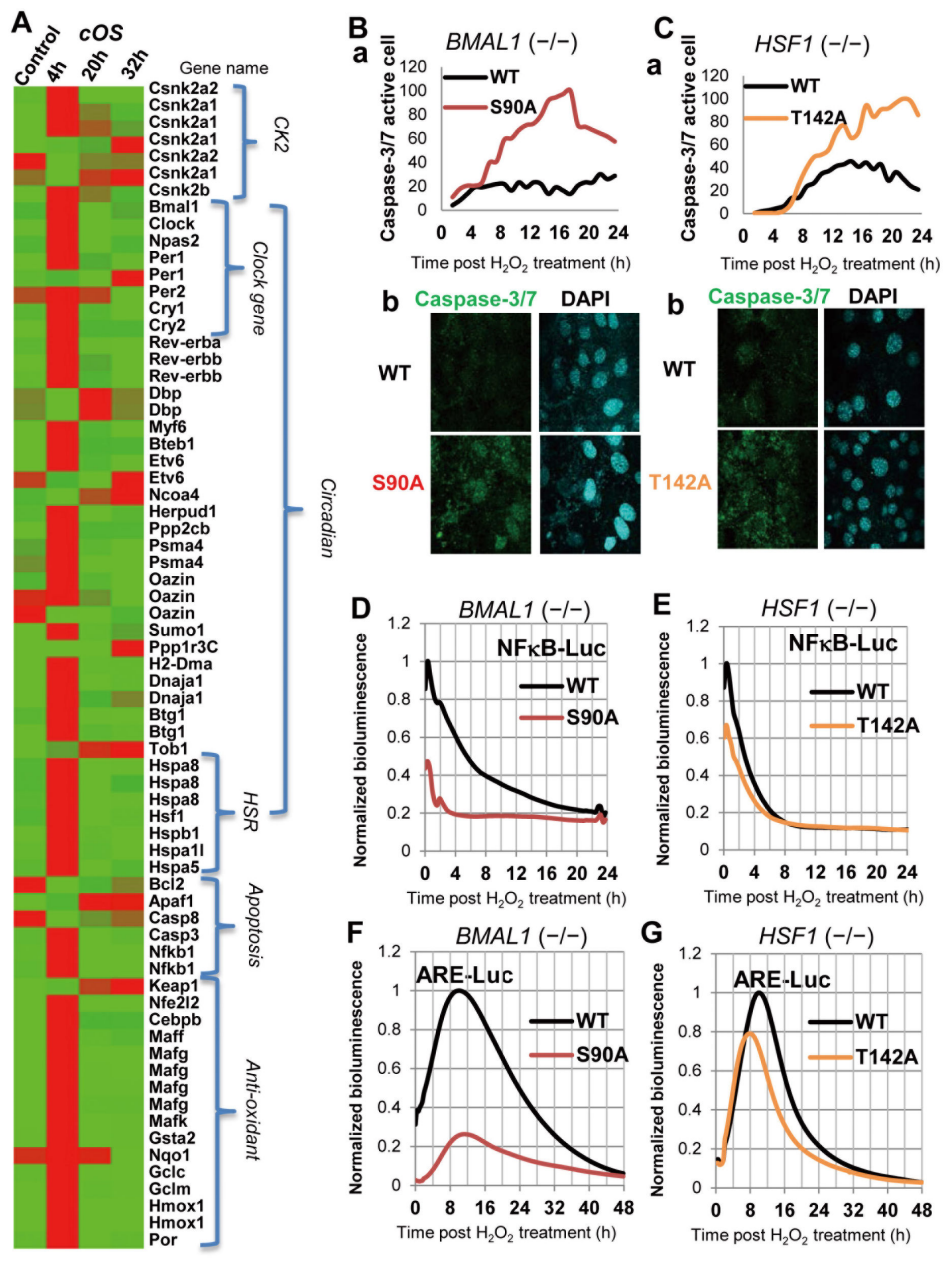
cOS-responsive circadian transcriptome regulated by CK2-signaling. (A) Expression profile of the core cOS-evoked circadian transcriptome for cell survival. Microarray analysis was performed to profile regulated genes in NIH-3T3:Per2-Luc with/without cOS-pulse. Functionally relevant genes for cOS-evoked responses were listed by annotation clustering and exploring biological pathways. A heatmap of the cOS-responsive genes encoding CK2, circadian, HSR, apoptosis, and anti-oxidant–related proteins is shown. Gradient representation from brightest red to brightest green indicates relatively high to low levels of gene expression. The values for the heatmap are shown in Table S2. (B-G) CK2-mediated BMAL1/HSF1 phosphorylation regulates anti-apoptotic/oxidant pathways after cOS-pulse. BMAL1^−/−^ MEFs harboring BMAL1-WT or BMAL1-S90A (B,D,F) and HSF1^−/−^ MEFs harboring HSF1-WT or HSF1-T142A (C,E,G) were cOS-pulsed. (B,C) (a) Normalized profiles for caspase-3/7-active cell numbers as monitored by live cell time-lapse imaging using CellEvent™ Caspase-3/7 Green (Movie S3 and S4). (b) Representative confocal images of fixed cells with DAPI-visualized nuclei post cOS-pulse are shown. (D,E) Cells transiently expressing NF-kappa-B-driven Luc were cOS-pulsed. Normalized profiles of NF-kappa-B-Luc are shown (n = 4). (F,G) Cells transiently expressing ARE-Luc, the Nrf2-mediated anti-oxidant reporter, were cOS-pulsed. Normalized profiles of ARE-Luc are shown (n = 4).

The circadian adaptive system is composed of a network of CK2-mediated signaling, circadian, and HSR systems for resetting clocks and cell survival in the presence of cOS by ROS (Figure S4). In order to understand the genome-wide molecular mechanisms that mediate this circadian adaptive system against ROS stress, we compared gene expression profiles in mouse fibroblasts (NIH3T3:Per2-Luc) with and without cOS-pulse (control; comparably weak resetting stimulus by fresh medium), using microarray. Gene expression levels were measured 4h (early stage after the immediate Per2-Luc surge), 20h and 32h after cOS-pulse. We identified up-regulated 3940 genes (10% of expressed probe sets; ≧2-fold) 4h after cOS-pulse vs. the control (Figure 5A), and 1051 genes (2.7% of expressed probe sets) with circadian fluctuations (Figure 5B). These data reveal global gene regulation at the early (post resetting) and circadian stage in the cOS-responsive circadian adaptive system. DAVID [26] was used to identify biological processes overrepresented within the cOS-up-regulated genes. Then, we sorted the identified annotation clusters (ACs) in several functional groups likely to be involved in cOS-responsive circadian adaptive system (Figure 5C, S5AB, S6 and Table S1). The functional ACs included circadian rhythm, response to heat/unfolded protein/radiation, oxidative stress-induced gene expression via Nrf2, and negative regulation of apoptosis/cell cycle. Genes in these sorted ACs are likely to contribute to cOS-triggered clock resetting and protective responses for cell survival. To verify this hypothesis by a different approach, we explored biological pathways using PathVisio [27]. We identified several biological pathways, similar to the above-mentioned ACs, which probably compose the core cOS-responsive circadian adaptive systems. Genes belonging to these pathways (CK2, Circadian, HSR, Apoptosis, and Anti-oxidant) displayed clustered up-regulation at 4h after cOS-triggered clock resetting, as observed on the heatmap (Figure 6A and Table S2). Several genes, including *Bmal1, NPAS2, Per1, Per2, Cry1, Dbp, Etv6, Ppp1r3C* (clock-related genes), *Hspa5* (HSR gene), *Gtsa2*, and *Nqo1* (anti-oxidant genes), exhibit circadian fluctuation associated with clock resetting. These genes/pathways are probably core components that are pivotal for clock resetting and cell survival in the cOS-responsive circadian transcriptome.

### CK2 Regulates Pro-survival Pathways against ROS Stress

To validate the notion that CK2-mediated signaling integrally controls these cOS-responsive pathways/transcriptome, as hypothesized above (Figures 6A and S4). We investigated whether apoptotic and anti-oxidant pathways are regulated by CK2-mediated BMAL1/HSF1 phosphorylation after cOS-pulse. We measured caspase-3/7 protease activity as a marker of early apoptosis [45] and NF-kappa-B-mediated gene expression as an anti-apoptotic marker [46]. NF-kappa-B transactivates genes encoding regulators of cellular survival. Notably, time-lapse imaging (Movie S3 and S4) revealed dramatically higher caspase-3/7 activity in MEFs harboring BMAL1-90A, with an evident surge after cOS pulse than in MEFs harboring BMAL1-WT (Figure 6Ba). Higher caspase-3/7 activity was also detected in MEFs harboring HSF1-T142A than in HSF1-WT harboring MEFs (Figure 6Ca). Consistently, confocal images showed similar caspase-3/7 activity patterns; higher caspase-3/7 activity was detected in MEFs harboring BMAL1-S90A and HSF1-T142A (Figure 6Bb, Cb). Moreover, BMAL1^−/−^ MEFs harboring BMAL1-WT exhibited remarkably higher NF-kappa-B-mediated transcriptional activity, demonstrated by NF- kappa-B-responsive promoter element-driven Luc, with an acute surge and gradual reduction to basal levels. A much less evident activity surge was observed in MEFs harboring BMAL1-90A (Figure 6D). Higher NF-kappa-B-Luc was also observed in MEFs harboring HSF1-WT than in those harboring HSF1-T142A (Figure 6E), but the difference was smaller than in the comparison of BMAL1-WT and S90A. H_2_O_2_ has consistently been implicated as an indirect activator of NF-kappa-B [47]. These temporal NF-kappa-B-Luc patterns, preceded by caspase-3/7 activity, are reasonable if NF-kappa-B suppresses caspase-3/7 activity due to NF-kappa-B-induced anti-apoptotic pathways. Supporting our present data, CLOCK is a positive regulator of NF-kappa-B-mediated transcription [48]. Intriguingly, given that CK2-mediated BMAL1-S90 phosphorylation is indispensable for nuclear accumulation and heterodimerization of CLOCK and BMAL1 as a core clock transactivator [16], BMAL1-S90 phosphorylation likely causes activation of NF-kappa-B-mediated transcription. FACS data have demonstrated larger differences in early apoptotic cells between WT and mutants and BMAL1-S90 phosphorylation is more crucial than HSF1-T142 phosphorylation in regulating the anti-apoptotic response after cOS-pulse. Given that BMAL1 and HSF1 elicit anti-oxidant responses to prohibit cellular damage [35,36], MEFs harboring BMAL1-WT/HSF-WT that survive after cOS-pulse are likely undamaged. To validate this anti-oxidant response, we examined gene expression mediated by Nrf2, one of the central transcription factors controlling antioxidant response [49]. BMAL1^−/−^ MEFs harboring BMAL1-WT exhibited remarkably higher Nrf2 -mediated transcriptional activity, with an evident acute surge and gradual decrease to basal levels 2 days after the cOS pulse. A much less evident surge was observed in MEFs harboring BMAL1-90A (Figure 6F). Higher ARE-Luc was observed in MEFs harboring HSF1-WT than in MEFs harboring HSF1-T142A (Figure 6G), but the difference was smaller than in the comparison of BMAL1-WT and S90A. These findings strongly suggest that CK2-mediated signaling integrally controls cOS-responsive anti-apoptotic and anti-oxidant pathways that lead to cell survival.

## Discussion

### Synergistic Clock Resetting and Cell Survival at the Branch Pont of Life and Death

**Figure 7:**
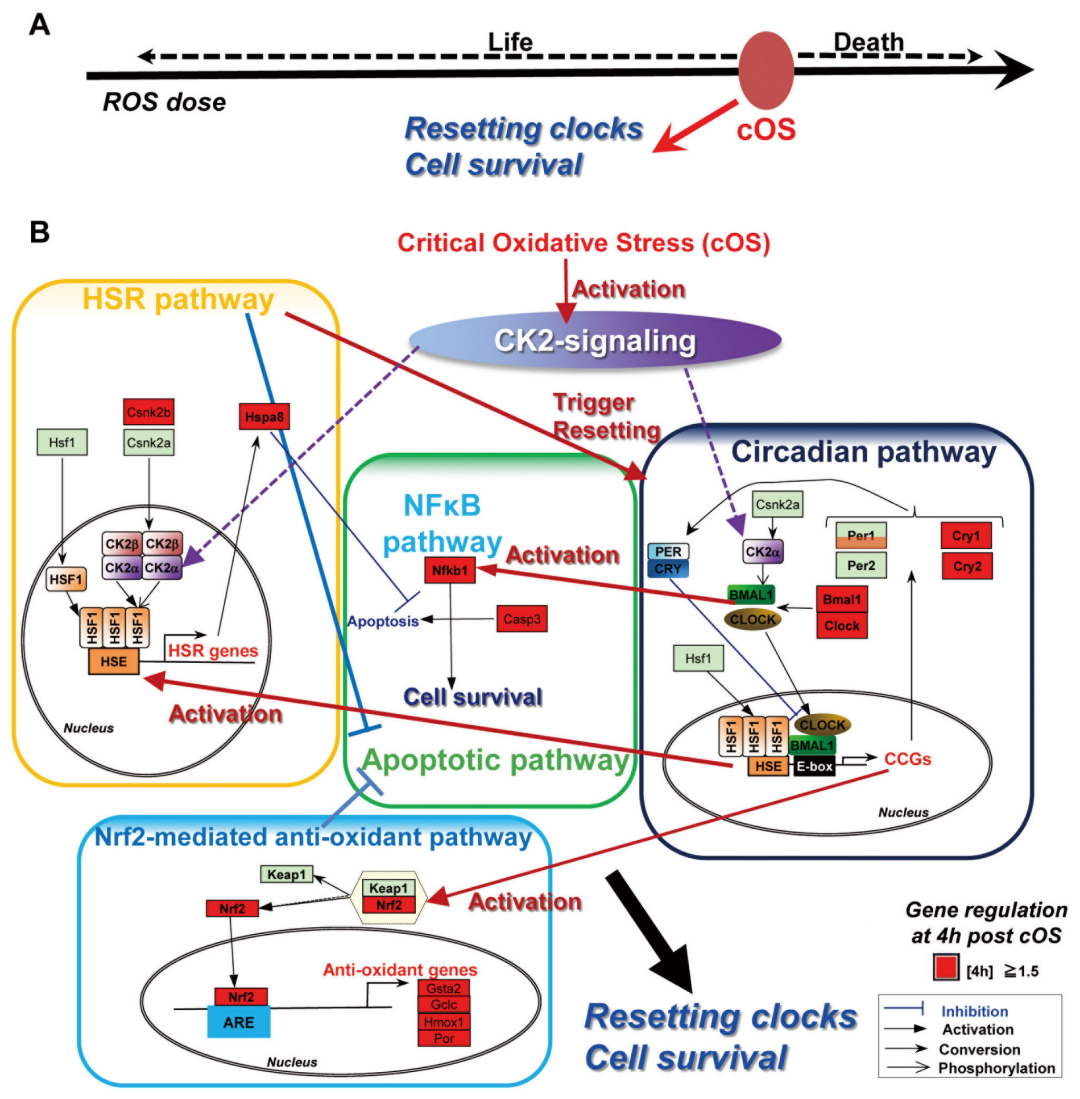
Circadian adaptive signaling responsive to the critical ROS stress. (A) The schematic figure shows resetting of the circadian clock by near-lethal doses of ROS (cOS) at the life-death boundary. (B) The schematic figure shows the core circadian signaling system for adaptation to critical ROS stress for cell survival. This signaling system is composed of CK2-orchestrated mutual crosstalk between circadian, HSR, apoptotic, and Nrf2-mediated anti-oxidant pathways. The component genes identified by PathVisio are shown as rectangles with red indicating up-regulation and pale green for unchanged expression at 4 h post cOS-pulse.

In this study, we identified ROS-dependent circadian control in mammals. We observed resetting of the circadian clock by near-lethal doses of ROS at the branch point of life and death (Figure 7A). This resetting process is accompanied by simultaneous activation of the cell survival systems. This kind of adaptation response brings biological order and homeostasis. We can also regard the resetting of multi-cellular clock phases as the transition process bringing order (synchronous state) from disorder (asynchronous state). This synchronous phase transition is presumably important for the multi-cellular adaptive response and cell survival. Here, we have demonstrated a direct correlation between circadian adaptation and life-survival phenomena. If this circadian adaptation system were not evolutionarily derived, the living species could not survive after exposure to this kind of ROS stress. Thus, we believe this synergistic clock resetting and cell survival implies a novel evolutionary aspect of the circadian system. 

### Circadian Adaptive Signaling Responsive to the Critical ROS Stress for Cell Survival

Transcriptome analysis elucidated the elaborate circadian-adaptive signaling system evoked by cOS (Figure 7B and S7). The BMAL1-controlled circadian system and HSF1-controlled HSR system are probably indispensable for this cOS-evoked pro-survival process, because ROS sensitivity is enhanced in BMAL1/HSF-deficient cells. By an unknown mechanism, intracellular ROS activates CK2 to phosphorylate HSF1 and BMAL1. Shorter-term phosphorylation of HSF1-T142 might cause immediate transactivation of *Per2*, monitored as acute Per2-Luc elevation, and other HSR genes, and BMAL1-HSF1 dimerization, to trigger clock resetting. Induced HSR genes, such as *Hspa1l*, *Hspa5* (*Grp78*) (Figure S7), and *Hspa8* (*Hsc70*) (Figure 7B) [50-52], as well as up-regulated *H2-DMa* and *Herpud1* in the circadian pathway [53,54] likely respond to unfolded proteins generated by ROS [55]. Interestingly, *Hspa1l*, an anti-inflammatory heat shock protein (Hsp) 70 gene, is positively associated with human survival [51]. Furthermore, these Hsp70 genes play anti-apoptotic roles by suppressing caspase-3 activity and formation of the apoptosome [56]. Up-regulated *Sumo1* (circadian pathway) and ubiquitin-related genes (Figure S7) may also contribute to the degradation of unfolded proteins [57]. Longer-term phosphorylation of BMAL1-S90 probably causes prolonged BMAL1-HSF1 dimerization to establish resetting of the core interlocked clock-feedback machinery that elicits subsequent transactivation of other CCGs such as *Per1, Cry1, Cry2, Rev-erb-alpha, Bmal1, Clock* and *NPAS2*. Interestingly, among CCGs, *Dbp* [58] was down-regulated (Figure S7). BMAL1 and CK2-controlled BMAL1-HSF1 dimerization may mediate activation of the HSR pathway by the circadian pathway. Supporting this, depletion of BMAL1 (Figure 2Ab) and abrogation of CK2-mediated phosphorylation (Figure 4Ab) resulted in remarkable impairment of the cOS-evoked HS-stress response. Due to mutagenesis-caused abrogation of CK2-mediated phosphorylation (Figures 3Be, 3C and 4), CK2-mediated BMAL1-S90/HSF1-T142 phosphorylation and BMAL1-HSF1 dimerization might be indispensable for cOS-evoked resetting and cell survival. In the HSR pathway, up-regulated *Hsbp1* may switch off HSR gene induction (Figure S7) [59]. Up-regulation of NF-kappa-B expression may strengthen this anti-apoptotic pathway for cell survival (Figure 7B). Our data do not exclude the contribution of NF-kappa-B activation through CK2-mediated phosphorylation-induced I-kappa-B degradation [60] to the cOS-responsive pathway. During the preparation of this manuscript, direct *Nrf2* transactivation via BMAL1:CLOCK binding to E-box in the promoter was reported [61]. Thus, in the circadian pathway, cOS-induced up-regulation of *Nrf2* via CK2-induced BMAL1:CLOCK-mediated transactivation results in the transactivation of anti-oxidant genes such as *Gsta2, Gclc, Hmox1*, and *Por*, encoding the anti-oxidant enzymes glutathione S-transferase-alpha2, glutamate-cysteine ligase catalytic subunit, heme oxigenase1, and P450 oxidoreductase, respectively (Figure 7B). Circadian adaptive activation of the Nrf2-mediated anti-oxidant pathway might remove ROS to suppress apoptosis. Our data do not exclude the contribution of the pathway via direct Nrf2 phosphorylation by CK2 [62]. Additionally, cOS-up-regulated genes encoding the heterodimeric partner of Nrf2 (*Maff, Mafg, Mafk*) [63,64], a transcription factor for the anti-oxidant genes (*Cebpb*) [65] (Figure 7B and S7) and other proteins in oxidative stress-responsive pathways (Figures S8), might contribute to cell survival. NF-kappa-B-mediated up-regulation *Sod1* and *Sod2* [66] after cOS-pulse is controlled by BMAL1 and CK2-mediated BMAL1-S90 phosphorylation, as evidenced by SOD1/2 quantitation after cOS-pulse (Figure S9) and the result in Figure 6D. This result (Figure S9) validates the link between the circadian system and pro-survival pathways under oxidative stress. Interestingly, peroxiredoxin, one of the removers of H_2_O_2_ with circadian enzymatic activity [67], was evidenced only by *Prdx1* and *Prdx6* up-regulation after cOS-pulse (Figure S7). Circadian fluctuations of large arrays of genes (Figures 5B, 6A, and S6) verify that cOS evokes not only immediate early gene regulation but also circadian gene regulation, confirming that this cOS-responsive signaling is circadian-adaptive. 

Based on the findings, we suppose that circadian system coordinates near-lethal ROS stress-induced pro-survival program through clock resetting-signaling. Our findings likely reveal a novel circadian-adaptive signaling system responsive to the critical ROS stress. The core part of this system is perhaps orchestrated by CK2. This system probably plays fundamental roles in protection against disease and death. We believe that molecular-targeted medicine against the components of this signaling system may aid the development of therapies and preventive measures against various ROS-inducible diseases such as cancer, metabolic disorders and neurodegenerative diseases.

## Supporting Information

Figure S1 **Determination of the appropriate dose for OS to reset circadian clocks.** NIH-3T3:Per2-Luc/HSE-SLR cells treated with various H_2_O_2_ doses as indicated. Relative (RLU) acute Per2-Luc/HSE-SLR (A) and normalized circadian Per2-Luc profiles (B) post-H_2_O_2_ treatment were monitored by real-time dual-color bioluminescence assay (n = 5). (C) Each relative cell survival score 1 week post H_2_O_2_ treatment is shown. The score ++++ indicates 90–100% viable (negative control level), +++ indicates 75–90% viable, ++ indicates 50–75% viable, + indicates 25–50% viable, − indicates less than 25% viable (in this case less than 5% viable).
(PDF)

Figure S2 **cOS resets single cellular circadian clocks.** U2OS:Per2-Luc/HSE-SLR cells were treated with various H_2_O_2_ doses as indicated (for 10min with 0.1-5mM, for 20min with 10mM). Temporal profiles of acute Per2-Luc/HSE-SLR surge (Aab), and circadian Per2-Luc (B) (n = 4) reveal synchronization of circadian Per2 rhythms following OS-pulse with an optimal dose similar to that of H_2_O_2_ treatment in NIH-3T3 cells. (C) Temporal Per2-Luc profiles of single cells (black dots represent average values), as monitored by time-lapse bioluminescence imaging (Movie S1B), showing synchronization of circadian Per2-Luc rhythms after cOS. (D) Each relative cell survival score 1 week post H_2_O_2_ treatment is shown.
(PDF)

Figure S3 **CK2 is pivotal to reset clocks and cell survival after cOS–pulse.** NIH-3T3:Per2-Luc/HSE-SLR were cOS-pulsed and treated with protein kinase inhibitors for CK2 (I; 25 microM DMAT, II; 25 microM TBCA), CK1 (100 microM CKI-7), JNK (10 microM L-JNKi1), p38 (10 microM SB203580), MEK (25 microM U0126) and PKA (5 microM inhibitor fragment (6-22) amide) as well as HSF1 inhibitor (100 microM KNK437) for the indicated duration (pre & post 1 h of cOS-pulse, added 1 h before the cOS-pulse, during the cOS-pulse, and 1 h after the cOS-pulse). Normalized acute Per2-Luc/HSE-SLR (A) and circadian Per2-Luc profiles (B) were monitored via real-time dual-color bioluminescence assay (n = 3). (C) Each relative cell survival score at 1 week following cOS-pulse is shown.
(PDF)

Figure S4 **CK2-signaling integrally controls cOS-evoked clock resetting and cell survival.** The schematic figure shows hypothetical crosstalk between CK2- mediated signaling, circadian, and HSR systems after cOS-pulse. The HSR system transmits cOS-evoked resetting information to the circadian system. The circadian system likely activates the HSR system. CK2 likely orchestrates the circadian and HSR systems through transactivation of CCGs and HSR genes, and BMAL1-HSF1 binding through BMAL1/HSF1 phosphorylation. Thus, CK2-mediated signaling integrally controls circadian resetting, which likely contributes to cell survival via circadian-HSR crosstalk.
(TIF)

Figure S5 **Functionally relevant ACs and categories of the up-regulated genes by cOS-pulse.** (A) A list of functionally relevant ACs are presented as a graph, showing numbers of genes (≧ 2-fold) included in the each AC. (B) The ACs were further sorted by their function. The graph shows numbers of genes included in the each functional category.
(PDF)

Figure S6 **Temporal expression profiles of genes included in the relevant ACs of the up-regulated genes.** Microarray analysis of gene expression in NIH-3T3:Per2-Luc with/without cOS-pulse was performed. A heatmap of several up-regulated genes included in the functionally relevant ACs to cOS-evoked responses is shown. Gradient representation from brightest red to brightest green indicates relatively high to low levels of gene expression. The values for the heatmap are shown in Table S1.
(PDF)

Figure S7 **cOS-responsive circadian adaptive signaling pathways.** The schematic shows the core circadian signaling system for adaptation to critical ROS stress for cell survival. This represents a detailed version of Figure 7B.
(PDF)

Figure S8 **Oxidative stress pathways containing up-regulated genes by cOS-pulse.** The component genes of an oxidative stress pathway identified using PathVisio are shown as rectangles with red indicating up-regulated, blue down-regulated, pale green unchanged, and gray for undetected, 4h post cOS-pulse.
(PDF)

Figure S9 **CK2-mediated BMAL1-S90 phosphorylation regulates SOD expression.** BMAL1^−/−^ MEFs (-), and BMAL1^−/−^ MEFs harboring BMAL1-WT or BMAL1-S90A MEFs were cOS-pulsed. At 8 h post with/ without (-) the treatment, cell lysates were analyzed by immunoblotting for SOD1 (using antibody; Upstate Biotechnology, USA), SOD2 (using antibody; Gene Tex, USA), actin and BMAL1. (A) Representative images are shown. (B) The immunoblot data were quantified by computerized densitometry as described previously [13]. A graph with error bar (±SD) showing normalized (to actin contents, and contents in WT) and averaged SOD1/2 contents (n = 4) at 8 h post cOS-pulse demonstrates significant differences between (-), WT and S90A: ⋆⋆ (P< 0.01), ⋆⋆⋆ (P< 0.001).
(PDF)

Table S1 **Expression profiles of genes belonging to the relevant annotation clusters.** Microarray analysis of gene expression in NIH-3T3:Per2-Luc with/without cOS-pulse was performed. AC, ID, name and value of the genes for the heatmap (Figure S6) are shown. Circadian fluctuated genes are highlighted blue (higher expression 32 h post cOS-pulse) or red (higher expression 20 h post cOS-pulse).
(PDF)

Table S2 **Expression profiles of genes in cOS-evoked signaling.** Microarray analysis of gene expression in NIH-3T3:Per2-Luc with/without cOS-pulse was performed. Gene name and value for the heatmap (Figure 6A) are shown. Acutely regulated and circadian fluctuated genes are highlighted with blue (higher expression in the control or at 32 h post cOS-pulse) or red (higher expression at 4 h or 20 h post cOS-pulse).
(PDF)

Movie S1 **A critical oxidative stress (cOS) for cell survival synchronizes circadian *Per2* rhythms in living single cells.** U2OS:Per2-Luc were cOS-pulsed after 2 days of pre- treatment (S1). Acute surges and circadian rhythms of Per2-Luc (pseudo-color) show synchronization of circadian *Per2* rhythms in each cell by cOS-pulse (S2), as monitored via time-lapse bioluminescence imaging analyzed using LV200 (Olympus, Japan). Note that only small parts of cells express Per2-Luc in the confluent cell culture.
(MP4)

Movie S2 **A critical oxidative stress (cOS) for cell survival synchronizes circadian *Per2* rhythms in living single cells.** U2OS:Per2-Luc were cOS-pulsed after 2 days of pre- treatment (S1). Acute surges and circadian rhythms of Per2-Luc (pseudo-color) show synchronization of circadian *Per2* rhythms in each cell by cOS-pulse (S2), as monitored via time-lapse bioluminescence imaging analyzed using LV200 (Olympus, Japan). Note that only small parts of cells express Per2-Luc in the confluent cell culture.
(MP4)

Movie S3 **Live imaging of Caspase-3/7 active cells after cOS-pulse in BMAL1^−/−^ MEFs harboring BMAL1-WT and BMAL1-S90A.** The movies show caspase-3/7active cells after cOS-pulse, and BMAL1^−/−^ MEFs harboring BMAL1-WT (S3) and BMAL1-S90A (S4) as monitored via time-lapse imaging fluorescence with CellEvent™ Caspase-3/7 Green (Molecular probes) analyzed using LV200 (Olympus).
(MP4)

Movie S4 **Live imaging of Caspase-3/7 active cells after cOS-pulse in BMAL1^−/−^ MEFs harboring BMAL1-WT and BMAL1-S90A.** The movies show caspase-3/7active cells after cOS-pulse, and BMAL1^−/−^ MEFs harboring BMAL1-WT (S3) and BMAL1-S90A (S4) as monitored via time-lapse imaging fluorescence with CellEvent™ Caspase-3/7 Green (Molecular probes) analyzed using LV200 (Olympus).
(MP4)
