# Supplementary figures and images for: ROS Stress Resets Circadian Clocks to Coordinate Pro-Survival Signals
Source: PLoS One. 2013 Dec 2;8(12):e82006. doi: 10.1371/journal.pone.0082006 (PMC3846904; doi:10.1371/journal.pone.0082006)

**A**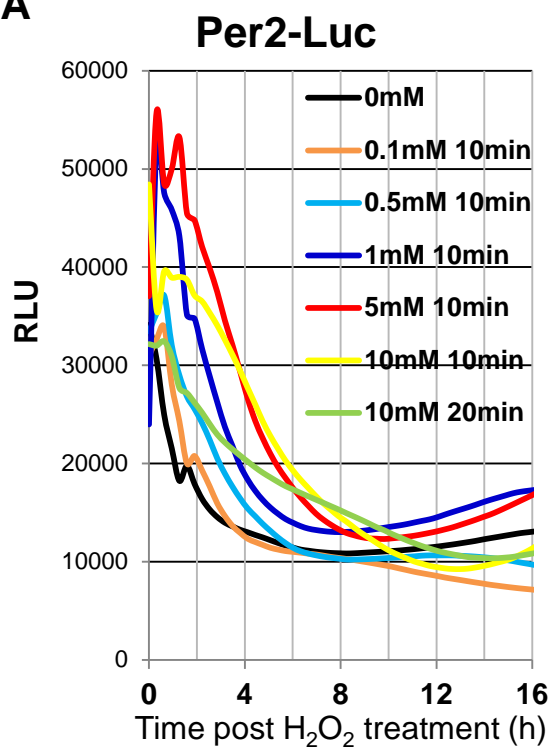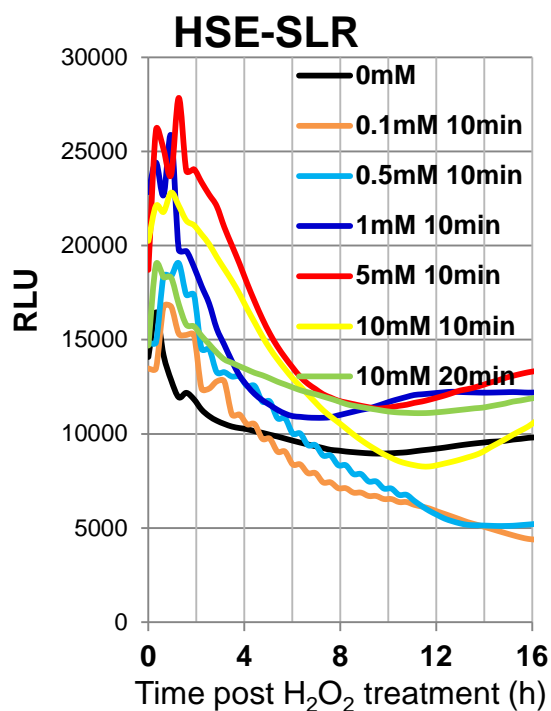**B**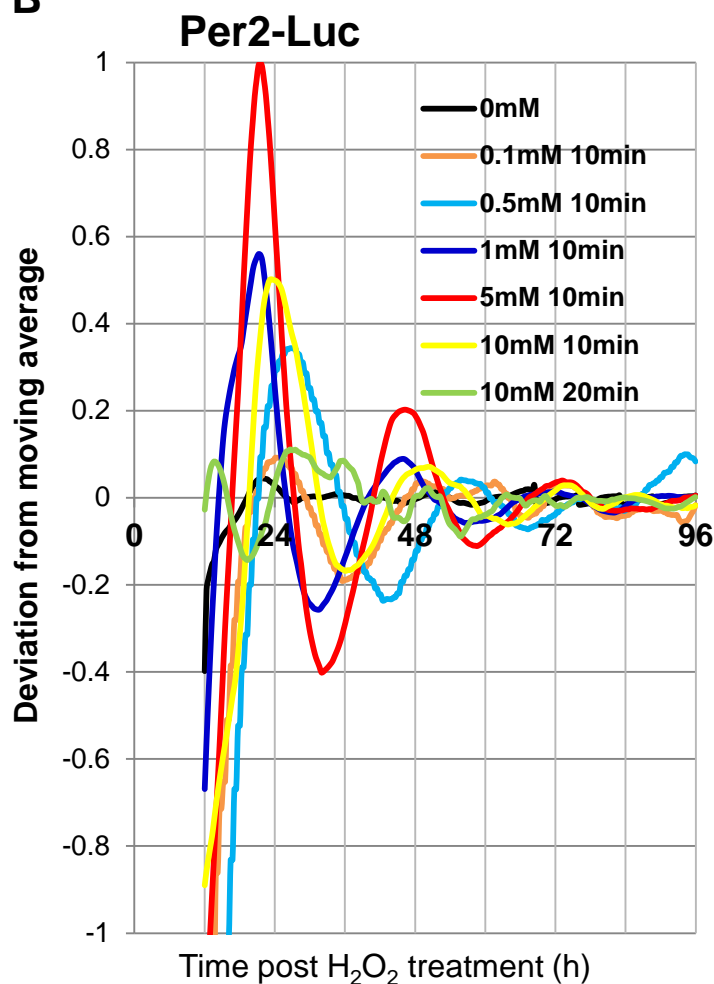**C**

|           | survival (1w) |
|-----------|---------------|
| (-)       | ++++          |
| 0.1mM 10m | ++++          |
| 0.5mM 10m | +++           |
| 1mM 10m   | +++           |
| 5mM 10m   | +++           |
| 10mM 5m   | +++           |
| 10mM 10m  | +             |
| 10mM 20m  | -             |

Supplement: Figure S1 — Determination of the appropriate dose for OS to reset circadian clocks. NIH-3T3:Per2-Luc/HSE-SLR cells treated with various H2O2 doses as indicated. Relative (RLU) acute Per2-Luc/HSE-SLR (A) and normalized circadian Per2-Luc profiles (B) post-H2O2 treatment were monitored by real-time dual-color bioluminescence assay (n = 5). (C) Each relative cell survival score 1 week post H2O2 treatment is shown. The score ++++ indicates 90–100% viable (negative control level), +++ indicates 75–90% viable, ++ indicates 50–75% viable, + indicates 25–50% viable, − indicates less than 25% viable (in this case less than 5% viable). (PDF) [file pone.0082006.s001.pdf]

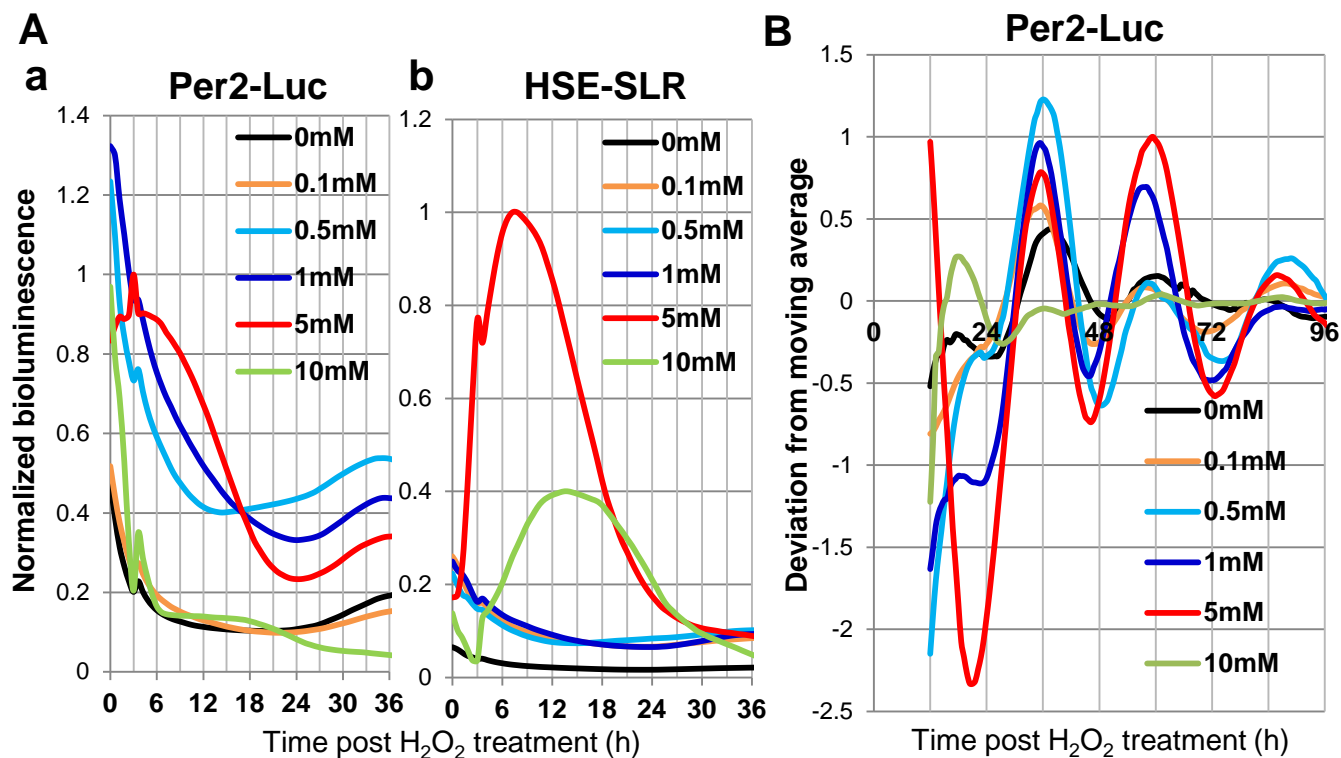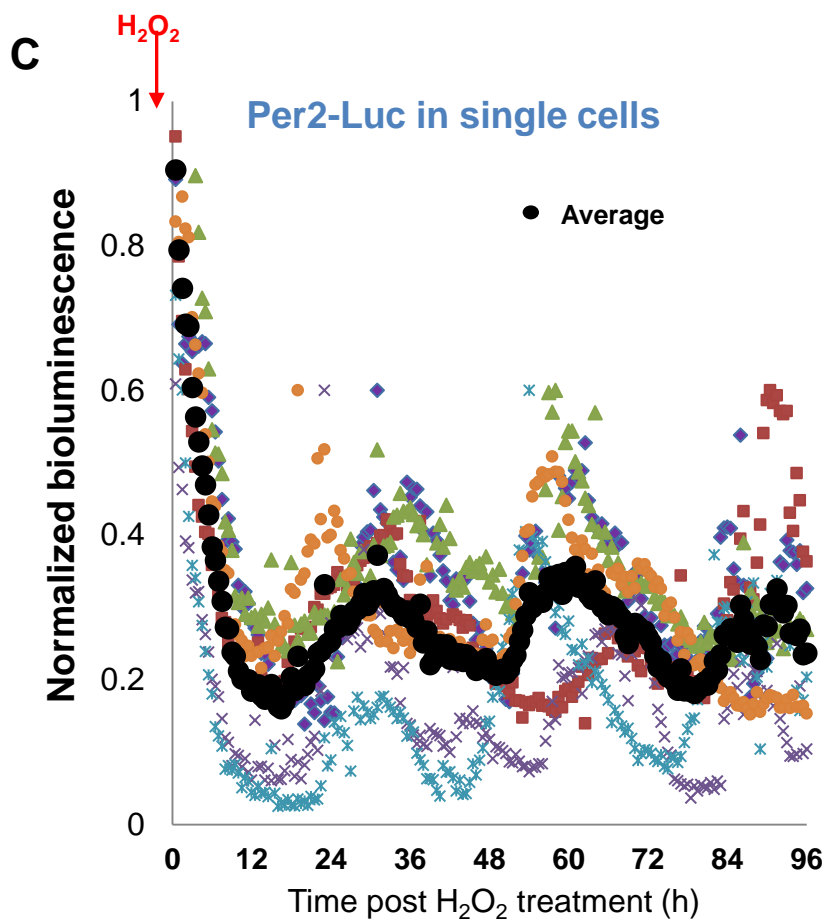

**D**

| survival (1w) |     |      |
|---------------|-----|------|
| (-)           |     | ++++ |
| 1mM           | 10m | +++  |
| 5mM           | 10m | +++  |
| 10mM          | 20m | -    |

Supplement: Figure S2 — cOS resets single cellular circadian clocks. U2OS:Per2-Luc/HSE-SLR cells were treated with various H2O2 doses as indicated (for 10min with 0.1-5mM, for 20min with 10mM). Temporal profiles of acute Per2-Luc/HSE-SLR surge (Aab), and circadian Per2-Luc (B) (n = 4) reveal synchronization of circadian Per2 rhythms following OS-pulse with an optimal dose similar to that of H2O2 treatment in NIH-3T3 cells. (C) Temporal Per2-Luc profiles of single cells (black dots represent average values), as monitored by time-lapse bioluminescence imaging (Movie S1B), showing synchronization of circadian Per2-Luc rhythms after cOS. (D) Each relative cell survival score 1 week post H2O2 treatment is shown. (PDF) [file pone.0082006.s002.pdf]

# A

Inhibitor: Pre 1h & Post 1h of  $H_2O_2$

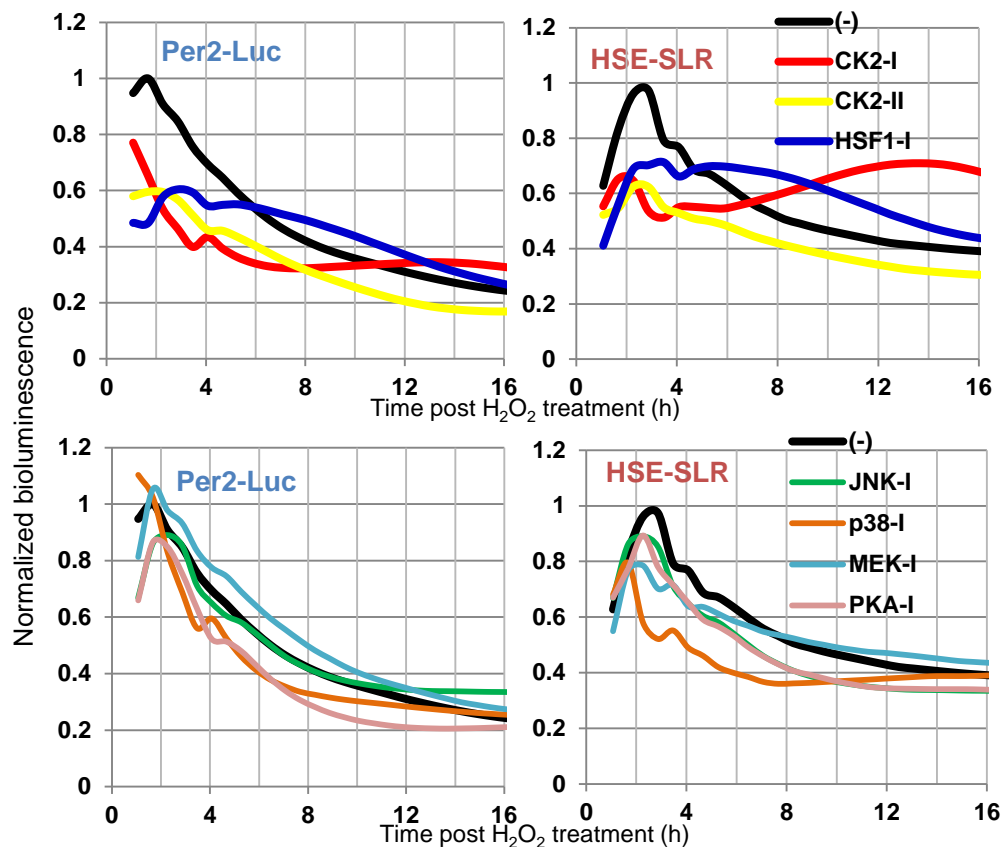

# C

Survival (1w)

|        |      |
|--------|------|
| (-)    | ++++ |
| CK2-I  | +    |
| CK2-II | +    |
| CK1-I  | ++++ |
| JNK-I  | ++++ |
| P38-I  | ++   |
| MEK-I  | ++++ |
| PKA-I  | ++++ |
| HSF1-I | +    |

# B

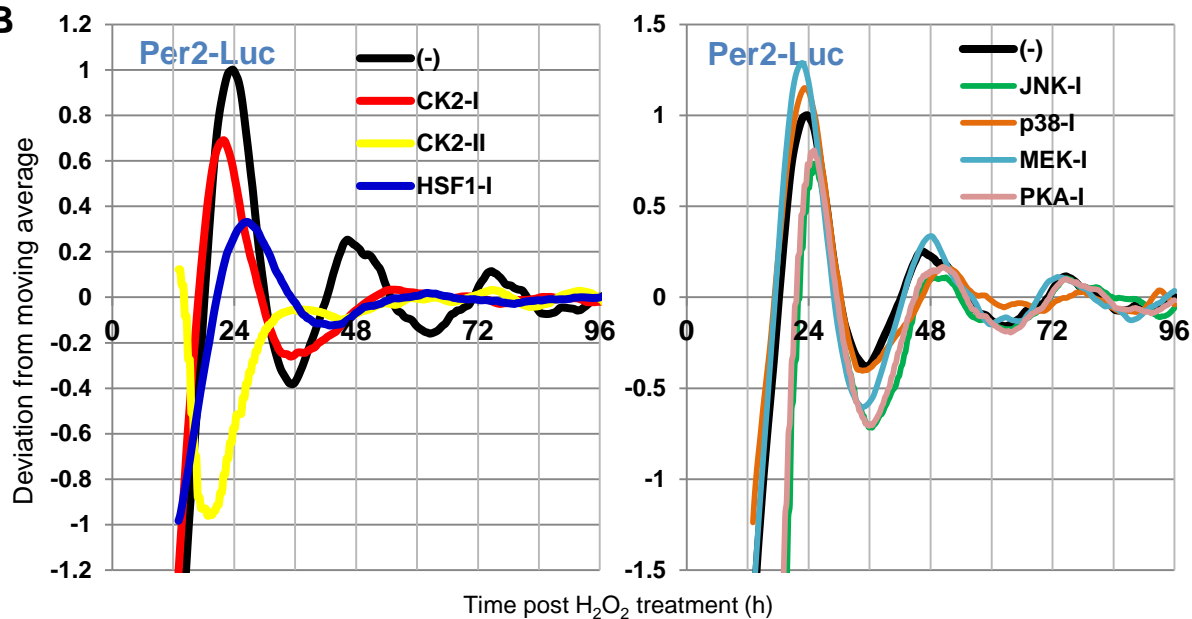

Supplement: Figure S3 — CK2 is pivotal to reset clocks and cell survival after cOS–pulse. NIH-3T3:Per2-Luc/HSE-SLR were cOS-pulsed and treated with protein kinase inhibitors for CK2 (I; 25 microM DMAT, II; 25 microM TBCA), CK1 (100 microM CKI-7), JNK (10 microM L-JNKi1), p38 (10 microM SB203580), MEK (25 microM U0126) and PKA (5 microM inhibitor fragment (6-22) amide) as well as HSF1 inhibitor (100 microM KNK437) for the indicated duration (pre & post 1 h of cOS-pulse, added 1 h before the cOS-pulse, during the cOS-pulse, and 1 h after the cOS-pulse). Normalized acute Per2-Luc/HSE-SLR (A) and circadian Per2-Luc profiles (B) were monitored via real-time dual-color bioluminescence assay (n = 3). (C) Each relative cell survival score at 1 week following cOS-pulse is shown. (PDF) [file pone.0082006.s003.pdf]

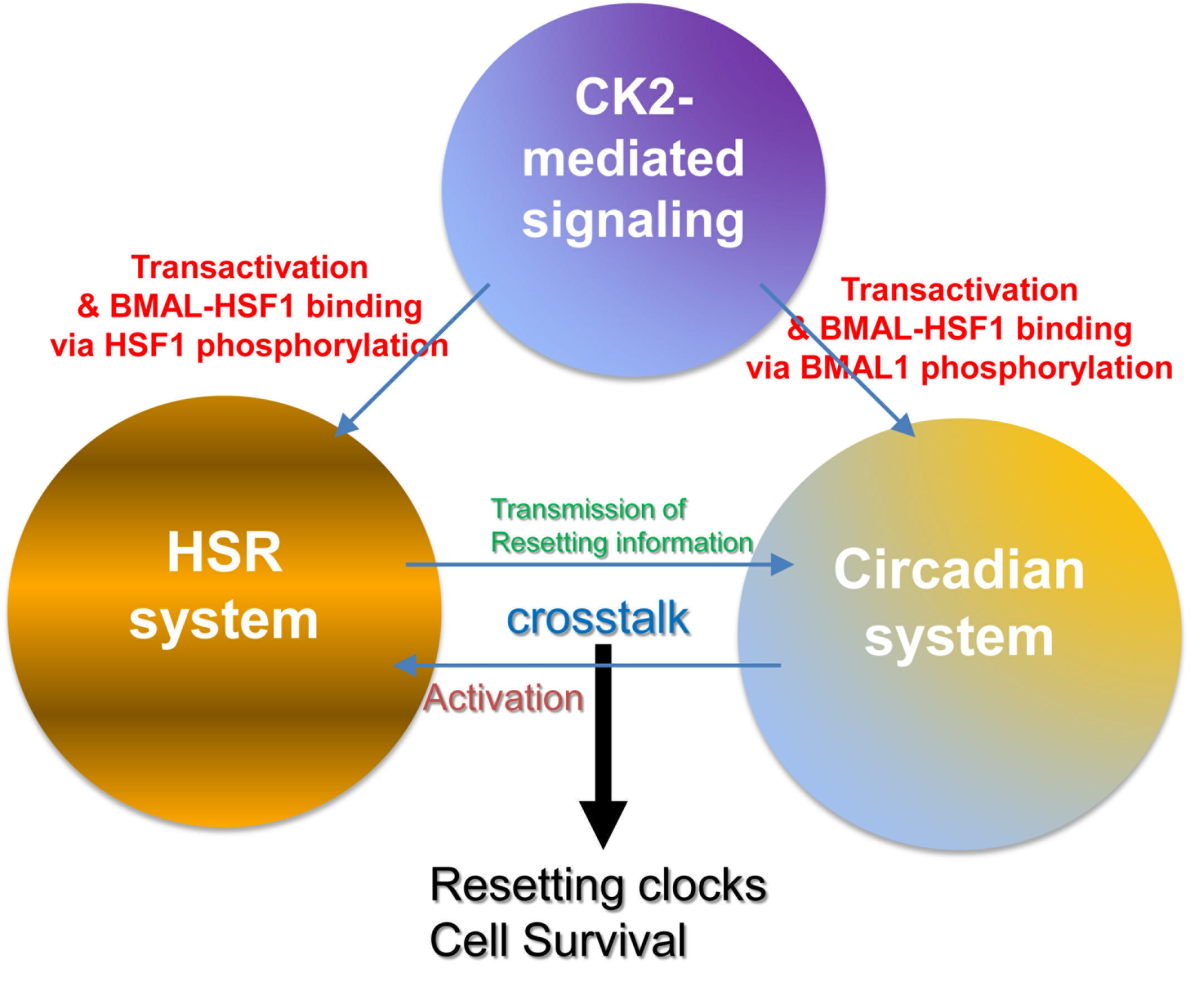

Supplement: Figure S4 — CK2-signaling integrally controls cOS-evoked clock resetting and cell survival. The schematic figure shows hypothetical crosstalk between CK2- mediated signaling, circadian, and HSR systems after cOS-pulse. The HSR system transmits cOS-evoked resetting information to the circadian system. The circadian system likely activates the HSR system. CK2 likely orchestrates the circadian and HSR systems through transactivation of CCGs and HSR genes, and BMAL1-HSF1 binding through BMAL1/HSF1 phosphorylation. Thus, CK2-mediated signaling integrally controls circadian resetting, which likely contributes to cell survival via circadian-HSR crosstalk. (TIF) [file pone.0082006.s004.tif]

## Up-regulated at 4h post cOS

**A**

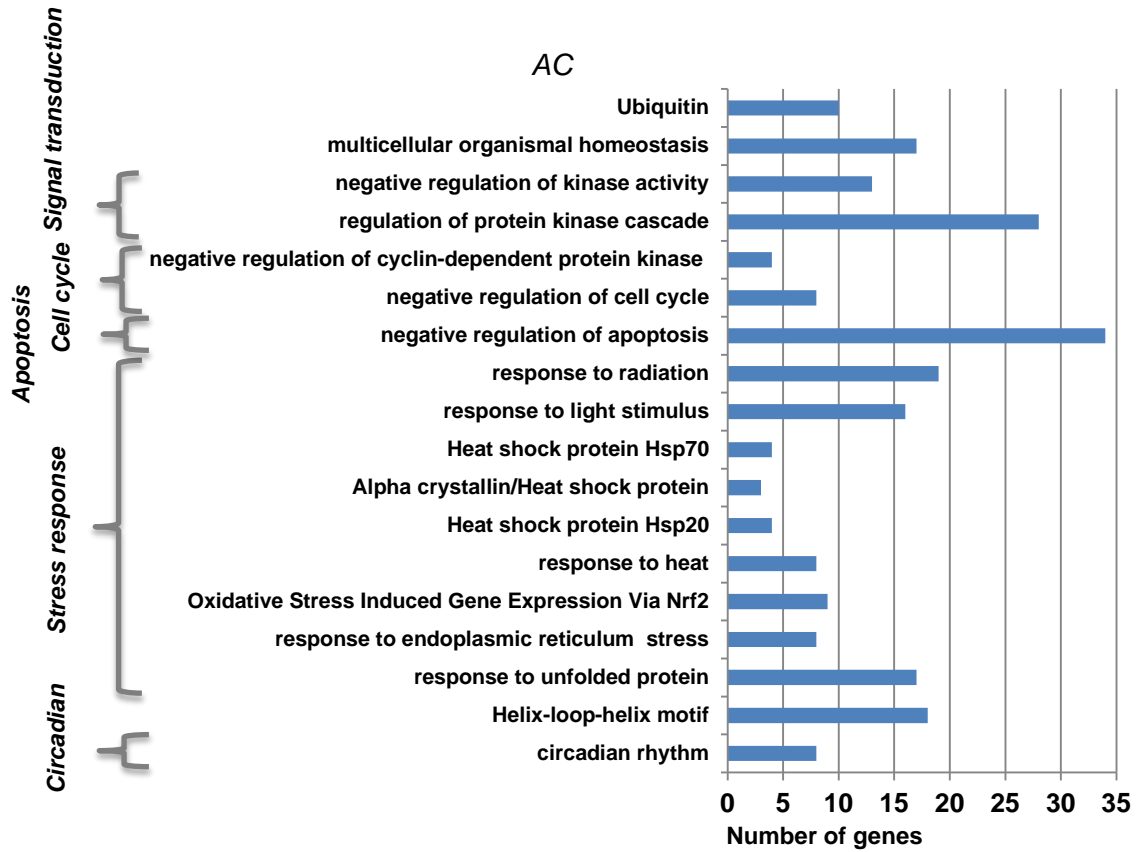

**B**

### Functional Category

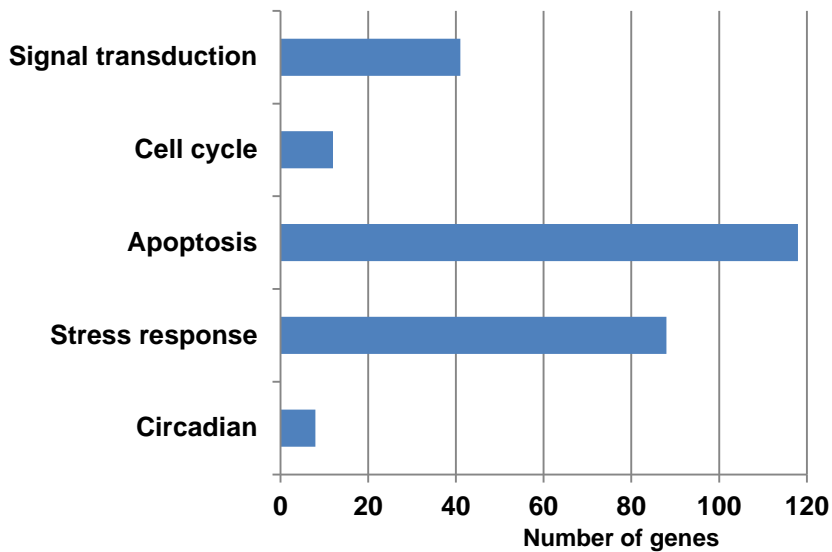

Supplement: Figure S5 — Functionally relevant ACs and categories of the up-regulated genes by cOS-pulse. (A) A list of functionally relevant ACs are presented as a graph, showing numbers of genes (≧ 2-fold) included in the each AC. (B) The ACs were further sorted by their function. The graph shows numbers of genes included in the each functional category. (PDF) [file pone.0082006.s005.pdf]

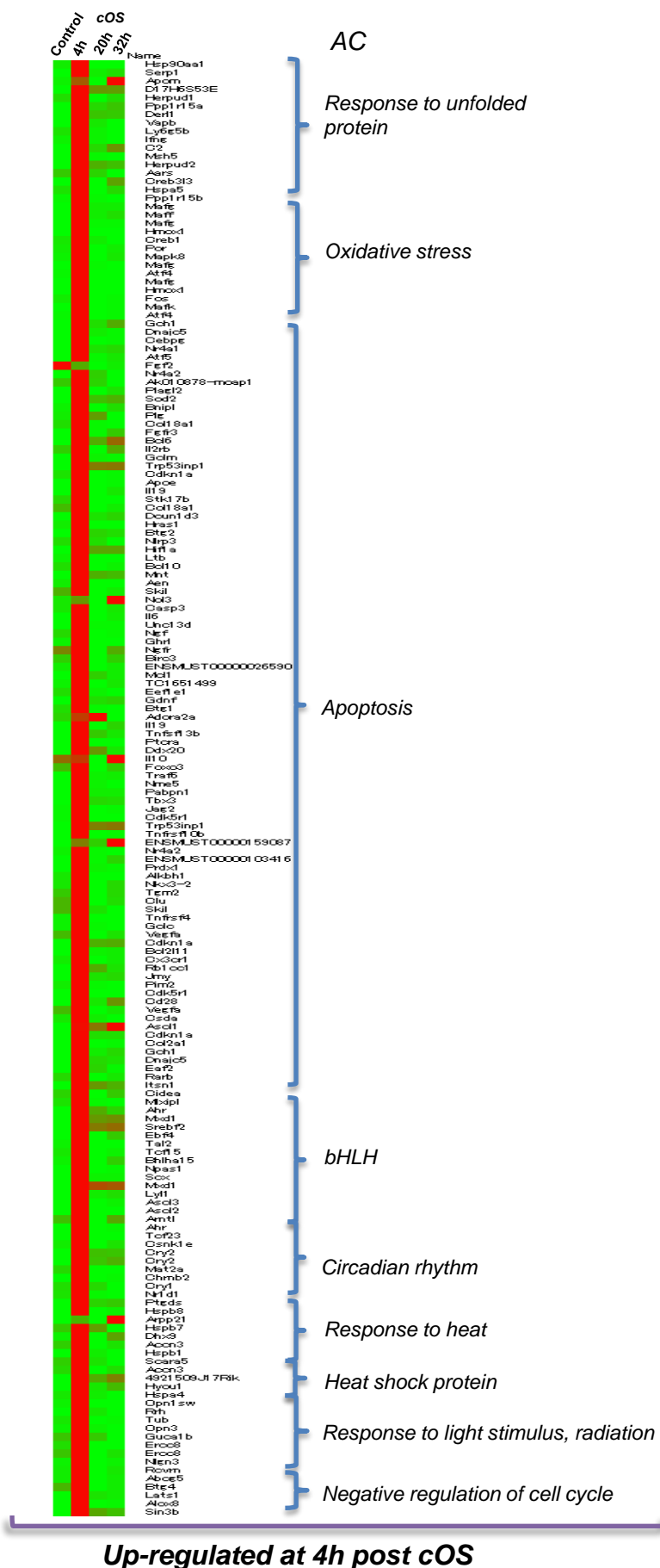

Supplement: Figure S6 — Temporal expression profiles of genes included in the relevant ACs of the up-regulated genes. Microarray analysis of gene expression in NIH-3T3:Per2-Luc with/without cOS-pulse was performed. A heatmap of several up-regulated genes included in the functionally relevant ACs to cOS-evoked responses is shown. Gradient representation from brightest red to brightest green indicates relatively high to low levels of gene expression. The values for the heatmap are shown in Table S1. (PDF) [file pone.0082006.s006.pdf]

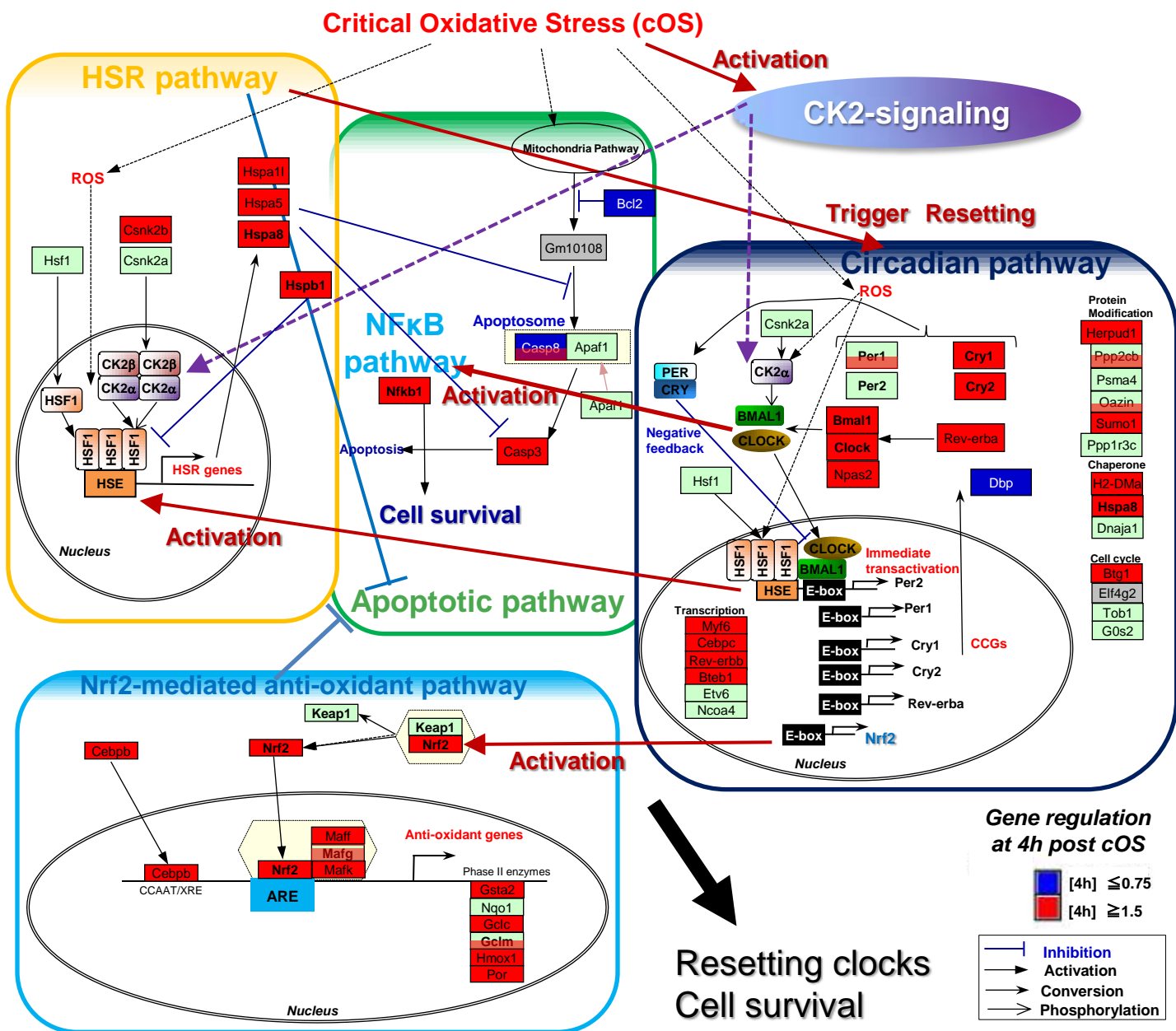

Supplement: Figure S7 — cOS-responsive circadian adaptive signaling pathways. The schematic shows the core circadian signaling system for adaptation to critical ROS stress for cell survival. This represents a detailed version of Figure 7B. (PDF) [file pone.0082006.s007.pdf]

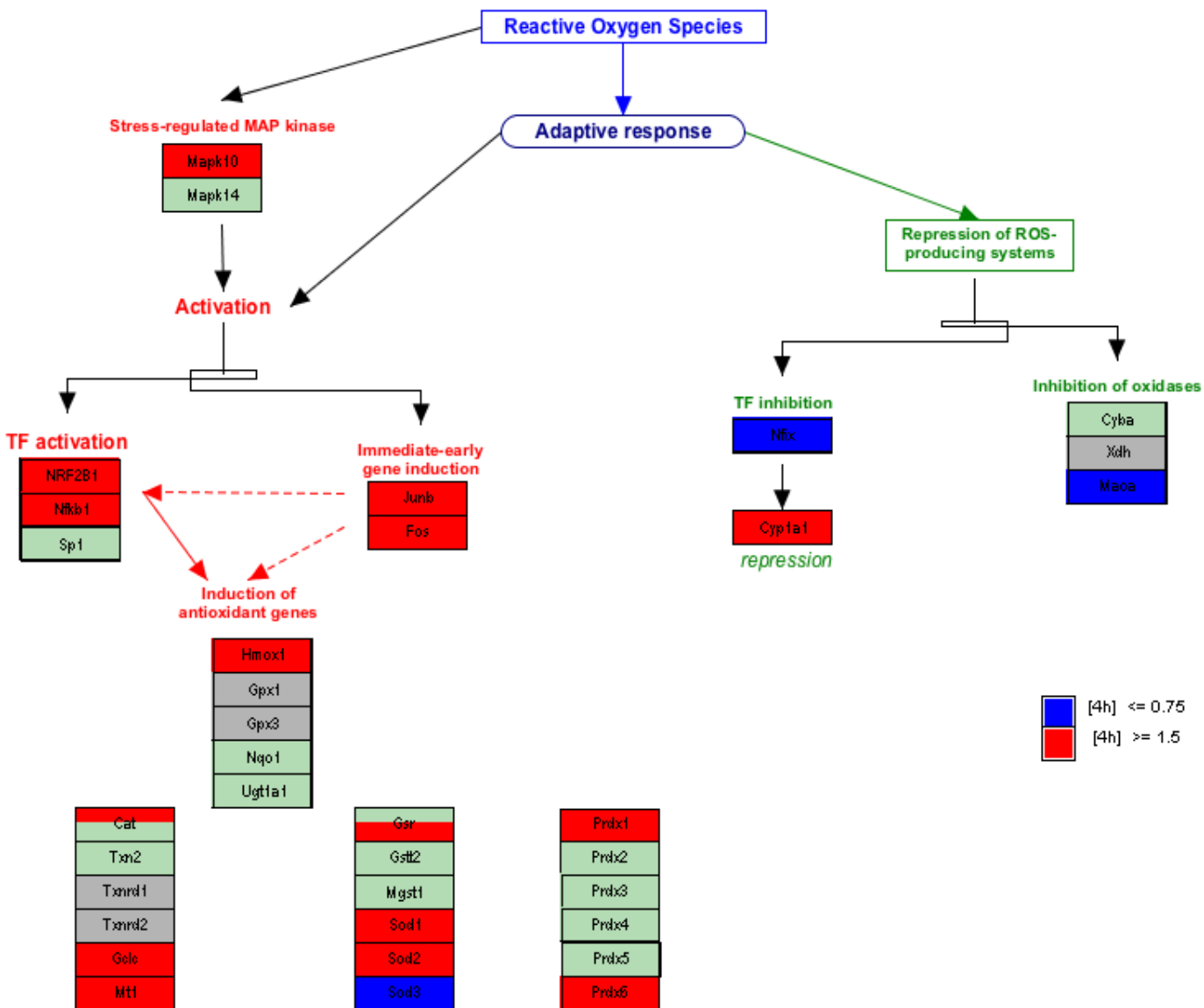

Supplement: Figure S8 — Oxidative stress pathways containing up-regulated genes by cOS-pulse. The component genes of an oxidative stress pathway identified using PathVisio are shown as rectangles with red indicating up-regulated, blue down-regulated, pale green unchanged, and gray for undetected, 4h post cOS-pulse. (PDF) [file pone.0082006.s008.pdf]

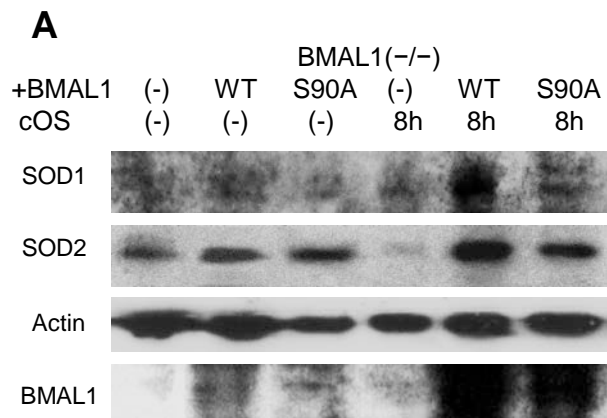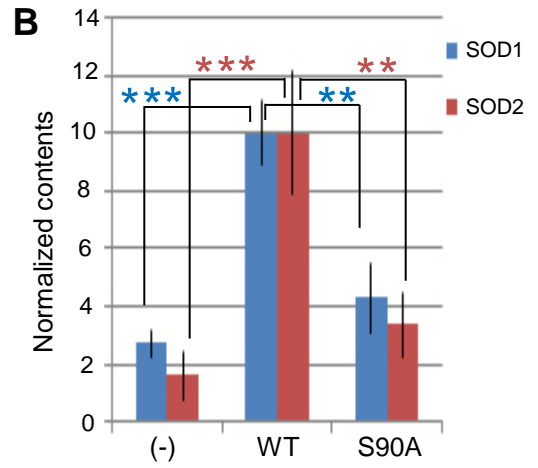

Supplement: Figure S9 — CK2-mediated BMAL1-S90 phosphorylation regulates SOD expression. BMAL1−/− MEFs (-), and BMAL1−/− MEFs harboring BMAL1-WT or BMAL1-S90A MEFs were cOS-pulsed. At 8 h post with/ without (-) the treatment, cell lysates were analyzed by immunoblotting for SOD1 (using antibody; Upstate Biotechnology, USA), SOD2 (using antibody; Gene Tex, USA), actin and BMAL1. (A) Representative images are shown. (B) The immunoblot data were quantified by computerized densitometry as described previously [13]. A graph with error bar (±SD) showing normalized (to actin contents, and contents in WT) and averaged SOD1/2 contents (n = 4) at 8 h post cOS-pulse demonstrates significant differences between (-), WT and S90A: ⋆⋆ (P< 0.01), ⋆⋆⋆ (P< 0.001). (PDF) [file pone.0082006.s009.pdf]
