# Supplementary material for: ROS Stress Resets Circadian Clocks to Coordinate Pro-Survival Signals
Source: PLoS One. 2013 Dec 2;8(12):e82006. doi: 10.1371/journal.pone.0082006 (PMC3846904; doi:10.1371/journal.pone.0082006)
Supplement: Table S1 — Expression profiles of genes belonging to the relevant annotation clusters. Microarray analysis of gene expression in NIH-3T3:Per2-Luc with/without cOS-pulse was performed. AC, ID, name and value of the genes for the heatmap (Figure S6) are shown. Circadian fluctuated genes are highlighted blue (higher expression 32 h post cOS-pulse) or red (higher expression 20 h post cOS-pulse). (PDF) [file pone.0082006.s010.pdf]

Table.S1 Expression profiles of genes belonging to the relevant annotation clusters.

cOS-4h	Cluster	ID	Name	Control	cOS-4h	cOs-20h	cOs-32h	4h vs cont	20h vs 32h
Up	AC8	A_55_P2057946	Hsp90aa1	26.36731	61.33132	23.4987645	23.61329	2.326036	0.99514986
Up	AC8	A_52_P520408	Serp1	3.669372	9.261045	4.10309256	4.410527	2.523878	0.93029521
Up	AC8	A_52_P150236	Apom	0.01759	0.089602	0.01260843	0.129334	5.094072	0.09748734
Up	AC8	A_51_P275751	D17H6S53	0.064985	0.149215	0.09903823	0.098551	2.296147	1.00494357
Up	AC8	A_51_P434101	Herpud1	3.0866	6.873791	2.53923972	3.069133	2.226978	0.82734755
Up	AC8	A_55_P2136880	Ppp1r15a	5.79568	17.30475	7.46964666	8.470225	2.985801	0.8818711
Up	AC8	A_51_P504522	Derl1	3.11235	7.161309	4.06104918	3.995394	2.300933	1.01643277
Up	AC8	A_52_P556140	Vapb	7.208405	15.55662	8.02569984	7.370174	2.158122	1.08894312
Up	AC8	A_55_P1972133	Ly6g5b	0.049459	0.156349	0.04240137	0.03623	3.161208	1.17032701
Up	AC8	A_52_P68893	Ifng	0.011763	0.061247	0.01202992	0.009427	5.206937	1.27616334
Up	AC8	A_51_P497985	C2	0.03062	0.093699	0.04062746	0.058293	3.060033	0.69695142
Up	AC8	A_51_P429865	Msh5	0.010999	0.114934	0.01313893	0.009109	10.44931	1.44235571
Up	AC8	A_51_P102860	Herpud2	0.637043	1.286006	0.83723516	0.795415	2.018712	1.05257715
Up	AC8	A_55_P2079291	Aars	0.019526	0.061086	0.01718259	0.008462	3.128511	2.03064662
Up	AC8	A_55_P2174987	Creb3l3	0.06464	0.238766	0.06134447	0.127368	3.693803	0.48163248
Up	AC8	A_55_P2076772	Hspa5	57.4354	148.2652	45.1037475	61.17975	2.581425	0.73723328
Up	AC8	A_52_P116372	Ppp1r15b	0.330888	1.052021	0.32494152	0.304363	3.179387	1.06761028
Up	AC15	A_55_P2047748	Mafg	6.302027	23.32222	7.54029203	7.444379	3.70075	1.01288396
Up	AC15	A_52_P608322	Maff	1.75527	4.301041	1.92542523	2.10585	2.450358	0.91432204
Up	AC15	A_66_P116632	Mafg	20.95441	64.92385	22.223487	22.55189	3.098338	0.98543799
Up	AC15	A_51_P263965	Hmox1	3.996638	39.04096	3.84818858	3.640142	9.768451	1.05715352
Up	AC15	A_52_P517730	Creb1	0.174872	0.425887	0.16604833	0.149407	2.435422	1.11137901
Up	AC15	A_55_P2029574	Por	10.97785	24.26837	10.0508039	10.91845	2.210668	0.92053399
Up	AC15	A_55_P1973299	Mapk8	0.00954	0.046004	0.00726867	0.010463	4.822171	0.69472812
Up	AC15	A_55_P2177026	Mafg	3.258451	10.89147	3.52595289	3.377795	3.342529	1.04386244
Up	AC15	A_55_P1979728	Atf4	69.20985	220.1977	65.9047571	67.59783	3.181595	0.97495376
Up	AC15	A_55_P2177023	Mafg	4.859171	15.52057	5.03507755	5.001993	3.194079	1.0066143
Up	AC15	A_55_P2029687	Hmox1	0.391591	2.93679	0.36483011	0.406271	7.499629	0.89799605
Up	AC15	A_52_P262219	Fos	0.02987	0.138729	0.02104981	0.025547	4.64448	0.82396965
Up	AC15	A_55_P1953241	Mafk	1.008209	3.399426	1.05495526	1.068461	3.371747	0.98735958
Up	AC15	A_51_P464238	Atf4	1.719418	6.088105	1.55320903	1.679443	3.540794	0.9248357
Up	AC27	A_52_P298002	Gch1	0.030985	0.087102	0.03810298	0.050047	2.811071	0.76133895
Up	AC27	A_55_P1982733	Dnajc5	0.652284	2.195762	0.76733857	0.686662	3.366268	1.11749162
Up	AC27	A_52_P438082	Cebpg	0.976975	3.47874	1.00324035	1.0375	3.560726	0.96697819
Up	AC27	A_51_P239654	Nr4a1	2.880898	7.961378	3.44756511	3.664112	2.763505	0.94090058
Up	AC27	A_55_P2105858	Atf5	40.97373	128.1007	43.9450359	49.28496	3.126411	0.89165209
Up	AC27	A_51_P399845	Fgf2	0.027703	0.020044	0.01625011	0.017148	0.723544	0.94765406
Up	AC27	A_52_P494622	Nr4a2	0.410427	0.833432	0.48319037	0.404568	2.030645	1.19433735
Up	AC27	A_55_P2007912	Ak010878-	0.046489	0.104568	0.04439402	0.031395	2.249311	1.41405506
Up	AC27	A_55_P1954161	Plagl2	0.049102	0.12114	0.04915078	0.057653	2.46711	0.85252734
Up	AC27	A_51_P172573	Sod2	122.0993	284.9378	162.135995	171.5328	2.333655	0.94521878
Up	AC27	A_55_P2091592	Bnipl	0.007834	0.049147	0.00601866	0.011426	6.273199	0.52673784
Up	AC27	A_52_P662013	Plg	0.011545	0.050791	0.02550551	0.008814	4.399495	2.89389846
Up	AC27	A_55_P2059352	Col18a1	0.037701	0.150187	0.02512937	0.022259	3.983696	1.12894977
Up	AC27	A_65_P06147	Fgfr3	0.019577	0.06887	0.02138597	0.029948	3.517887	0.71409771
Up	AC27	A_52_P161495	Bcl6	1.71931	3.577695	2.33103732	2.836165	2.08089	0.82189762
Up	AC27	A_51_P286496	Il2rb	0.019163	0.060247	0.00982462	0.026342	3.143929	0.3729654
Up	AC27	A_51_P320089	Gclm	0.563401	1.803172	0.61313319	0.592479	3.200512	1.03486068
Up	AC27	A_55_P1973906	Trp53inp1	0.078667	0.214602	0.15181034	0.153135	2.727973	0.99135089
Up	AC27	A_55_P1986282	Cdkn1a	0.065432	0.255879	0.04235271	0.046742	3.910622	0.90609549
Up	AC27	A_51_P171999	Apoe	0.460437	3.02104	0.49736414	0.525076	6.561248	0.94722348
Up	AC27	A_55_P2128144	Il19	0.050181	0.375325	0.0452839	0.076538	7.479501	0.59165398
Up	AC27	A_51_P214197	Stk17b	1.068686	2.619007	0.78313253	0.775445	2.450681	1.00991346
Up	AC27	A_55_P2124791	Col18a1	0.046352	0.138344	0.01177732	0.021116	2.984671	0.55774207
Up	AC27	A_52_P503361	Dcun1d3	0.076731	0.179961	0.08827262	0.094993	2.34537	0.92925039
Up	AC27	A_66_P139159	Hras1	0.056633	0.116411	0.05164198	0.052281	2.055523	0.98778489
Up	AC27	A_52_P31543	Btg2	0.131118	0.389231	0.17435501	0.166094	2.968562	1.04973698
Up	AC27	A_55_P2273439	Nlrp3	0.011972	0.03723	0.01241453	0.008439	3.109826	1.47113771
Up	AC27	A_55_P2149011	Hif1a	0.034633	0.092766	0.05551654	0.055185	2.678515	1.00601408
Up	AC27	A_51_P302358	Ltb	0.040048	0.751279	0.00611671	0.008339	18.75943	0.73347043
Up	AC27	A_51_P209527	Bcl10	3.388254	7.413975	2.82373989	3.084823	2.188141	0.91536517
Up	AC27	A_52_P477431	Mnt	9.432283	20.27898	12.761821	12.43715	2.149954	1.02610474
Up	AC27	A_55_P2145804	Aen	20.92834	42.91484	19.8492199	19.39645	2.050561	1.02334308
Up	AC27	A_51_P118132	Skil	0.986185	1.797453	0.61821313	0.700933	1.822633	0.88198657
Up	AC27	A_55_P2135311	Nol3	0.032877	0.07107	0.02319369	0.137809	2.161686	0.16830283
Up	AC27	A_52_P117576	Casp3	0.113615	0.246419	0.10033939	0.113127	2.168902	0.8869588
Up	AC27	A_55_P1997756	Il6	0.798655	8.069252	0.84670011	1.242172	10.10355	0.68162857
Up	AC27	A_55_P1976928	Unc13d	0.030085	0.268786	0.035977	0.035505	8.934173	1.0132871
Up	AC27	A_55_P2074337	Ngf	1.435041	4.647505	0.89049815	1.01432	3.238586	0.87792642
Up	AC27	A_55_P2022399	Ghrl	0.055887	0.315348	0.04412743	0.045425	5.642628	0.97143058
Up	AC27	A_52_P236448	Ngfr	0.035773	0.053258	0.01919406	0.030507	1.488758	0.62917336
Up	AC27	A_55_P1980287	Birc3	0.777094	1.561414	0.60258854	0.793052	2.009299	0.75983439
Up	AC27	A_51_P128320	ENSMUST	0.00868	0.040013	0.00656086	0.009006	4.609823	0.72851314
Up	AC27	A_52_P93933	Mcl1	2.283456	5.743419	2.96704128	2.491059	2.51523	1.19107635
Up	AC27	A_55_P2061243	TC165149	0.050561	0.120163	0.04630132	0.052517	2.376585	0.88164245
Up	AC27	A_51_P164420	Eef1e1	1.633212	3.543456	1.460519	1.3297	2.169624	1.09838264
Up	AC27	A_55_P2045896	Gdnf	6.229471	17.49638	7.65934753	8.055805	2.808646	0.95078609
Up	AC27	A_55_P2181191	Btg1	1.93433	5.433085	1.55991155	1.416479	2.808769	1.1012602
Up	AC27	A_55_P2109382	Adora2a	0.027009	0.085589	0.10861416	0.010963	3.168898	9.90693509
Up	AC27	A_55_P2001489	Il19	0.023072	0.144995	0.02536985	0.04291	6.284471	0.59123784
Up	AC27	A_51_P257934	Tnfsf13b	0.013652	0.108292	0.03417564	0.022082	7.932442	1.54767709
Up	AC27	A_55_P2122884	Ptcra	0.009179	0.301973	0.00692707	0.009813	32.89723	0.70587714
Up	AC27	A_65_P07038	Ddx20	0.042765	0.129307	0.0782146	0.050077	3.023651	1.56187877
Up	AC27	A_51_P430766	Il10	0.014953	0.016941	0.00896804	0.019111	1.132937	0.46926475
Up	AC27	A_55_P2129309	Foxo3	0.063605	0.136809	0.03620455	0.054516	2.150925	0.66410414
Up	AC27	A_52_P510215	Traf6	0.291237	0.632477	0.30822022	0.317119	2.171693	0.97193834
Up	AC27	A_55_P1966102	Nme5	0.069648	0.155708	0.07337522	0.068221	2.235638	1.07554844
Up	AC27	A_55_P2064442	Pabpn1	14.50049	29.57034	15.5549192	15.60059	2.039265	0.99707256
Up	AC27	A_55_P1969002	Tbx3	22.29411	45.15145	25.521298	25.17617	2.025263	1.01370846
Up	AC27	A_51_P451338	Jag2	0.007395	0.052541	0.0058343	0.007681	7.104733	0.75959326
Up	AC27	A_55_P2173183	Cdk5r1	0.260285	1.309842	0.22427414	0.23873	5.032336	0.93944754
Up	AC27	A_51_P175580	Trp53inp1	1.658082	5.352966	3.56049074	3.701679	3.228409	0.96185831
Up	AC27	A_55_P2027836	Tnfrsf10b	0.660363	3.332021	0.63640232	0.724121	5.045743	0.87886184
Up	AC27	A_55_P2037236	ENSMUST	0.014273	0.040241	0.02401214	0.066039	2.819457	0.36360658
Up	AC27	A_55_P1983508	Nr4a2	0.105042	0.220553	0.10279477	0.098277	2.099675	1.04597096
Up	AC27	A_55_P2021084	ENSMUST	0.018478	0.041534	0.01939607	0.022363	2.247755	0.86732704
Up	AC27	A_55_P2147427	Prdx1	37.08649	93.40484	39.4667961	39.15499	2.518568	1.00796336
Up	AC27	A_52_P522023	Alkbh1	0.325197	1.069729	0.24800883	0.24854	3.289475	0.99786116
Up	AC27	A_55_P2177113	Nkx3-2	0.024325	0.07634	0.01855451	0.027469	3.138328	0.67546054
Up	AC27	A_52_P220879	Tgm2	0.023923	0.053479	0.01627417	0.0217	2.235454	0.74996933
Up	AC27	A_55_P2017929	Clu	0.016729	0.04066	0.0069461	0.011371	2.430504	0.61084405
Up	AC27	A_52_P597461	Skil	0.096575	0.216313	0.06899656	0.049439	2.23983	1.39559584
Up	AC27	A_55_P2125491	Tnfrsf4	0.502599	1.24253	0.46814966	0.480714	2.47221	0.97386257
Up	AC27	A_51_P365019	Gclc	2.932332	10.82289	2.80135166	2.556718	3.690882	1.0956828
Up	AC27	A_52_P638895	Vegfa	11.4859	26.27405	5.74420445	8.167069	2.287505	0.70333728
Up	AC27	A_55_P1976204	Cdkn1a	0.037451	0.217923	0.09174533	0.098332	5.818947	0.93301702
Up	AC27	A_51_P484880	Bcl2l11	0.08626	0.256761	0.09330822	0.09672	2.976587	0.96472897
Up	AC27	A_52_P99810	Cx3cr1	0.008143	0.039446	0.0060635	0.008436	4.843971	0.71873234
Up	AC27	A_66_P119418	Rb1cc1	0.654515	1.649151	0.97466566	0.758376	2.519652	1.28520166
Up	AC27	A_51_P441469	Jmy	0.125022	0.286523	0.14403783	0.149335	2.291776	0.96452671
Up	AC27	A_52_P608255	Pim2	0.039936	0.091317	0.03849931	0.036591	2.286601	1.05215387
Up	AC27	A_55_P2156583	Cdk5r1	0.763341	4.080116	0.69883308	0.714543	5.345074	0.9780146
Up	AC27	A_55_P1979491	Cd28	0.020649	0.061054	0.02392776	0.037844	2.9568	0.63227391
Up	AC27	A_52_P249424	Vegfa	2.23147	5.736517	0.97398037	1.386202	2.570735	0.70262536
Up	AC27	A_55_P1960566	Csda	15.81159	34.29102	15.5838307	14.01236	2.168726	1.11214893
Up	AC27	A_51_P437327	Ascl1	0.038479	0.041129	0.03997775	0.041202	1.068883	0.97028737
Up	AC27	A_51_P363947	Cdkn1a	1.311818	15.27316	3.1315135	3.208954	11.64275	0.97586731
Up	AC27	A_55_P2004179	Col2a1	0.009833	0.163721	0.00739951	0.01053	16.65015	0.70270472
Up	AC27	A_55_P2106175	Gch1	0.333346	2.987403	0.53102515	0.708806	8.961855	0.7491826
Up	AC27	A_55_P2093720	Dnajc5	0.288236	0.804908	0.36276076	0.349235	2.792534	1.03872885
Up	AC27	A_55_P2102624	Eaf2	0.01572	0.458666	0.09017298	0.028112	29.17669	3.20765504
Up	AC27	A_55_P2084807	Rarb	0.022727	0.125103	0.00603158	0.016816	5.504703	0.35868129
Up	AC27	A_55_P1994613	Itsn1	0.010622	0.039058	0.02209001	0.019338	3.677295	1.14229446
Up	AC27	A_51_P199168	Cidea	0.028513	0.082283	0.01778804	0.030978	2.885815	0.57421065
Up	AC42	A_55_P2119957	Mlxipl	0.057922	0.151757	0.05043456	0.047324	2.620038	1.065723
Up	AC42	A_55_P2109613	Ahr	3.443167	7.101023	4.63819026	4.089264	2.062352	1.13423588
Up	AC42	A_55_P2025248	Mxd1	1.334517	3.82919	2.35962222	2.475283	2.869345	0.95327383
Up	AC42	A_55_P2000487	Srebf2	0.064811	0.153224	0.1124534	0.116676	2.364166	0.96381169
Up	AC42	A_55_P2011981	Ebf4	0.02779	0.072338	0.0280901	0.037103	2.603021	0.75707614
Up	AC42	A_52_P385594	Tal2	0.038574	0.165573	0.03201836	0.033696	4.292352	0.95020467
Up	AC42	A_51_P508580	Tcf15	0.012872	0.069186	0.00715391	0.01025	5.374801	0.69796887
Up	AC42	A_52_P340669	Bhlha15	0.011473	0.053567	0.0091912	0.017576	4.668814	0.52293343
Up	AC42	A_55_P2031721	Npas1	0.008261	0.109309	0.00632516	0.008988	13.23247	0.70371726
Up	AC42	A_51_P380432	Scx	0.025725	0.573554	0.04871909	0.051543	22.29523	0.94521243
Up	AC42	A_51_P490795	Mxd1	0.640698	1.671452	1.3826078	1.335678	2.608797	1.03513573
Up	AC42	A_51_P143190	Lyl1	0.01546	0.118019	0.02219865	0.02842	7.633597	0.781105
Up	AC42	A_55_P2185553	Ascl3	0.009306	0.272927	0.01139592	0.020296	29.32718	0.56147901
Up	AC42	A_55_P2033725	Ascl2	0.021357	0.381843	0.01523054	0.014331	17.87922	1.06277412
Up	AC42	A_55_P2408588	Arntl	0.934502	1.961272	0.58580861	1.060768	2.098735	0.55224966
Up	AC42	A_65_P06603	Ahr	0.009812	0.045503	0.01007384	0.010582	4.637627	0.95198979
Up	AC42	A_52_P300451	Tcf23	0.007871	0.162681	0.00607377	0.00862	20.66724	0.70461979
Up	AC64	A_66_P117366	Csnk1e	0.052257	0.197453	0.04075845	0.058152	3.778471	0.70089197
Up	AC64	A_55_P2016540	Cry2	2.2314	5.341193	2.99097137	2.928896	2.393651	1.02119417
Up	AC64	A_55_P1953708	Cry2	0.398182	0.85128	0.49917438	0.536264	2.137918	0.93083655
Up	AC64	A_51_P500344	Mat2a	24.06636	49.13428	19.6528141	19.693	2.041616	0.99795938
Up	AC64	A_52_P222026	Chrnb2	0.008552	0.134758	0.00682616	0.011171	15.75698	0.61103685
Up	AC64	A_52_P420500	Cry1	0.039706	0.113721	0.04110643	0.028286	2.864113	1.45326782
Up	AC64	A_51_P223776	Nr1d1	0.188338	0.400531	0.16759055	0.162718	2.12666	1.029946
Up	AC64	A_55_P2100928	Ptgds	0.025656	0.068182	0.03253876	0.034537	2.657561	0.94214414
Up	AC67	A_51_P464387	Hspb8	5.659629	18.28613	5.70156985	6.23812	3.230976	0.91398843
Up	AC67	A_55_P2016937	Arpp21	0.007316	0.043281	0.00584208	0.132838	5.916084	0.04397891
Up	AC67	A_51_P346445	Hspb7	0.017057	0.043603	0.02294076	0.010065	2.556365	2.27920391
Up	AC67	A_55_P2351505	Dhx9	0.100753	0.265063	0.09238456	0.156633	2.630811	0.58981671
Up	AC67	A_55_P2162727	Accn3	0.038027	0.085918	0.02677397	0.033695	2.259386	0.79459559
Up	AC67	A_55_P2039284	Hspb1	0.007385	0.052661	0.0104746	0.007711	7.131067	1.35835201
Up	AC67	A_55_P1982400	Scara5	0.019325	0.043527	0.01699089	0.013868	2.252418	1.22521166
Up	AC67	A_66_P132496	Accn3	0.03886	0.089097	0.02950438	0.037837	2.292755	0.77977419
Up	AC83	A_55_P2190152	4921509J'	0.197762	0.469455	0.28790557	0.335115	2.373837	0.85912562
Up	AC83	A_65_P20167	Hyou1	0.058479	0.129175	0.06443883	0.078261	2.208901	0.82338399
Up	AC83	A_55_P2054157	Hspa4	21.07493	49.59655	20.1382721	18.23022	2.353344	1.10466429
Up	AC100	A_55_P1965886	Opn1sw	0.015823	0.154777	0.00932343	0.009831	9.781825	0.94838543
Up	AC100	A_55_P2071334	Rrh	0.008519	0.049849	0.01237619	0.00888	5.851224	1.3936845
Up	AC100	A_51_P262238	Tub	0.01327	0.086958	0.00618411	0.008541	6.553166	0.72405259
Up	AC100	A_52_P215176	Opn3	0.065301	0.181141	0.06471953	0.058859	2.773945	1.09956356
Up	AC100	A_55_P1963906	Guca1b	0.016312	0.039524	0.01568647	0.009411	2.422904	1.66685291
Up	AC100	A_51_P341010	Ercc8	1.309939	2.716821	1.09826504	1.130622	2.074005	0.97138146
Up	AC100	A_52_P471227	Ercc8	0.106437	0.226508	0.06963768	0.088244	2.128102	0.78914975
Up	AC100	A_55_P1960994	Nlgn3	0.007232	0.042127	0.00575581	0.007567	5.824816	0.76066103
Up	AC100	A_66_P102910	Rcvrn	0.013714	0.092345	0.00733314	0.015732	6.733754	0.46614141
Up	AC100	A_55_P2031288	Abcg5	0.00812	0.038976	0.00613357	0.008407	4.800147	0.72958795
Up	AC105	A_51_P491190	Btg4	0.015239	0.036295	0.00627383	0.008566	2.381781	0.73242706
Up	AC105	A_52_P642801	Lats1	0.317464	0.850757	0.3718491	0.356174	2.679854	1.04400947
Up	AC105	A_55_P2029957	Alox8	0.007651	0.106537	0.00600567	0.008179	13.92486	0.73431277
Up	AC105	A_55_P2072543	Sin3b	0.024611	0.062007	0.03790262	0.033851	2.519455	1.11970323
