# Supplementary material for: ROS Stress Resets Circadian Clocks to Coordinate Pro-Survival Signals
Source: PLoS One. 2013 Dec 2;8(12):e82006. doi: 10.1371/journal.pone.0082006 (PMC3846904; doi:10.1371/journal.pone.0082006)
Supplement: Table S2 — Expression profiles of genes in cOS-evoked signaling. Microarray analysis of gene expression in NIH-3T3:Per2-Luc with/without cOS-pulse was performed. Gene name and value for the heatmap (Figure 6A) are shown. Acutely regulated and circadian fluctuated genes are highlighted with blue (higher expression in the control or at 32 h post cOS-pulse) or red (higher expression at 4 h or 20 h post cOS-pulse). (PDF) [file pone.0082006.s011.pdf]

Table.S2 Expression profiles of genes in cOS-evoked signaling

	Probe-Gene name (alternative description)	Control	cOS-4h	cOS-20h	cOS-32h	4h vs control	20h vs 32h
1	A_66_P117655 - Csnk2a2 (CK2a', Casein kinase 2 alpha-prime catalytic subunit)	0.022107007	0.102096577	0.016753752	0.016498355	4.618290346	1.015480149
2	A_55_P2022845 - Csnk2a1 (CK2a, Casein kinase 2 alpha catalytic subunit)	4.428198824	5.999354989	5.239524175	4.651122701	1.354807051	1.126507407
3	A_51_P397768 - Csnk2a1 (CK2a, Casein kinase 2 alpha catalytic subunit)	0.643116719	0.721190786	0.693481619	0.673480229	1.121399529	1.029698556
4	A_55_P1971828 - Csnk2a1 (CK2a, Casein kinase 2 alpha catalytic subunit)	0.501161797	0.503072645	0.509257197	0.524515205	1.003812837	0.970910266
5	A_52_P207614 - Csnk2a2 (CK2a', Casein kinase 2 alpha-prime catalytic subunit)	1.462757687	1.077797115	1.26887396	1.280179516	0.736825466	0.991168773
6	A_55_P2022840 - Csnk2a1 (CK2a, Casein kinase 2 alpha catalytic subunit)	3.774392613	3.071731041	4.013786557	4.301583969	0.813834531	0.933095015
7	A_51_P373379 - Csnk2b (CK2b, Casein kinase 2 beta regulatory subunit)	10.2129405	15.47623354	13.02776774	11.50548584	1.515355302	1.132309224
8	A_55_P2408588 - Arntl (BMAL1, Brain muscle arnt-like protein)	0.934501808	1.961271633	0.585808609	1.060767706	2.09873498	0.552249664
9	A_52_P334670 - Clock	1.064832655	1.672356358	1.045277276	1.082123305	1.57053444	0.965950249
10	A_52_P534749 - Npas2 (neuronal PAS domain protein 2)	0.008166072	0.01660074	0.006289421	0.008722999	2.032891701	0.721015903
11	A_55_P1970033 - Per1 (Period1)	1.687263621	2.339634389	1.902384361	1.70348117	1.386644244	1.116762776
12	A_55_P2019327 - Per1 (Period1)	0.014364285	0.02162663	0.010338537	0.058210774	1.505583466	0.177605214
13	A_51_P282760 - Per2 (Period2)	0.038414261	0.044614949	0.039301931	0.023498851	1.161416303	1.672504371
14	A_52_P420500 - Cry 1(Cryptochrome1)	0.039705608	0.113721347	0.041106433	0.028285518	2.864112974	1.453267817
15	A_55_P2016540 - Cry2 (Cryptochrome2)	2.231400381	5.341192941	2.990971368	2.928895865	2.393650636	1.021194165
16	A_51_P223776 - Nr1d1 (Rev-erb-alpha)	0.188338102	0.400531052	0.167590549	0.162717802	2.126659703	1.029945998
17	A_55_P2148534 - Nr1d2 (Rev-erb-beta)	1.580534799	2.893102826	2.044634827	1.57913225	1.830458164	1.294783782
18	A_52_P303891 - Nr1d2 (Rev-erb-beta)	0.805971539	1.689534839	0.963866427	0.741074686	2.096271093	1.300633317
21	A_55_P2032081 - Dbp (D site albumin promoter binding protein)	0.951097244	0.375084617	1.542301584	0.977207468	0.394370417	1.578274455
22	A_55_P2032079 - Dbp (D site albumin promoter binding protein)	0.915865144	0.400449244	1.495995878	0.947904991	0.437236035	1.578212893
23	A_51_P386794 - Myf6 (myogenic factor 6)	0.009600689	0.017909576	0.011023921	0.009715177	1.865446949	1.134711287
24	A_65_P19780 - Bteb1(Klf9 Kruppel-like factor 9)	19.20736888	34.84841174	23.43206183	22.94765773	1.814325114	1.021109087
25	A_55_P2230913 - Etv6 (ets variant gene 6 (TEL oncogene))	0.53472693	0.729647658	0.568136682	0.54216151	1.364523866	1.047910395
26	A_55_P2000404 - Etv6 (ets variant gene 6 (TEL oncogene))	0.010589186	0.009092382	0.008162505	0.011298868	0.85864787	0.722417945
27	A_55_P2016862 - Ncoa4(nuclear receptor coactivator 4)	4.718137293	4.572982189	5.030869267	5.24568581	0.969234659	0.959048912
28	A_51_P434101 - Herpud1(HERP; Mif1)	3.08659993	6.873791063	2.539239719	3.069133072	2.226978299	0.827347547
29	A_55_P1994913 - Ppp2cb (protein phosphatase 2, catalytic subunit, beta isozyme)	5.118301735	6.716249847	5.464496228	5.412994792	1.312202796	1.009514407
30	A_65_P09443 - Psma4 (proteasome subunit, alpha type 1)	8.535616747	9.828103835	7.991173623	7.778252129	1.151422812	1.027373951
31	A_52_P81468 - Psma4 (proteasome subunit, alpha type 1)	6.049184653	6.89307494	5.375735666	5.352757891	1.139504799	1.004292698
32	A_51_P234544 - Oazin (Azin1 antizyme inhibitor 1)	5.481340662	8.59994361	5.175165499	4.664210823	1.568948938	1.109547938
33	A_52_P24631 - Oazin (Azin1 antizyme inhibitor 1)	1.551837936	1.620173033	1.355871475	1.157899998	1.044034944	1.17097459
34	A_52_P117313 - Oazin (Azin1 antizyme inhibitor 1)	0.059195159	0.045866362	0.045410194	0.048265679	0.774832989	0.940838189
35	A_55_P2002155 - Sumo1 (SMT3 suppressor of mif two 3 homolog 1)	27.44914677	41.23967334	28.34171195	32.57932029	1.502402741	0.869929504
36	A_52_P30451 - Ppp1r3c (protein phosphatase 1, regulatory subunit 3C)	0.009161177	0.007668151	0.006653674	0.176526221	0.837026836	0.03769227
37	A_51_P473734 - H2-Dma (histocompatibility 2, class II, locus Dma)	0.076891184	0.910324819	0.113418501	0.13255999	11.83913125	0.855601309
38	A_55_P2087984 - Dnaja1 (DnaJ (Hsp40) homolog, subfamily A, member 1)	30.21618674	48.14119337	30.51240515	31.51467763	1.593225306	0.968196645
39	A_55_P1992149 - Dnaja1 (DnaJ (Hsp40) homolog, subfamily A, member 1)	0.075011573	0.098667783	0.081880499	0.086621418	1.31536747	0.945268513
40	A_55_P2181191 - Btg1 (B-cell translocation gene 1, anti-proliferative)	1.934329675	5.433084556	1.559911552	1.416478643	2.808768653	1.101260199
41	A_55_P2098598 - Btg1 (B-cell translocation gene 1, anti-proliferative)	87.76732305	168.7255914	97.15515222	92.84478677	1.922419251	1.046425498
42	A_51_P293339 - Tob1 (transducer of ErbB-2.1)	0.445706992	0.566674638	0.701704491	0.75654407	1.271406211	0.927513041
43	A_52_P12877 - Hspa8 (heat shock protein 8, Hsc70)	12.12040667	21.2414817	10.12129509	10.54129859	1.752538696	0.96015638
44	A_55_P2080603 - Hspa8 (heat shock protein 8, Hsc70)	146.4024615	197.552049	135.7020778	153.1221307	1.349376554	0.886234257
45	A_55_P2069721 - Hspa8 (heat shock protein 8, Hsc70)	68.96469075	111.3798929	62.26732511	63.77060221	1.615027802	0.976426801
46	A_55_P2039284 - Hspb1 (heat shock factor binding protein 1)	0.007384785	0.052661397	0.010474603	0.007711258	7.131067052	1.358352035
47	A_55_P2035593 - Hsf1(Heat shock factor-1)	2.607223029	3.789790767	2.35594082	2.450844072	1.453573678	0.961277319
48	A_55_P2039284 - Hspb1 (heat shock factor binding protein 1)	0.007384785	0.052661397	0.010474603	0.007711258	7.131067052	1.358352035
49	A_51_P340747 - Hspa1l (HSP70-1L, heat shock 70kDa protein 1-like)	0.010919889	0.033411024	0.012183643	0.00991201	3.059648683	1.229179854
50	A_55_P2076772 - Hspa5 (heat shock 70kDa protein 5)	57.43539987	148.2651789	45.10374746	61.17974973	2.581425031	0.737233278
51	A_51_P380013 - Bcl2 (B-cell CLL/lymphoma 2)	0.236162598	0.136766557	0.162146692	0.186905807	0.57912031	0.86753159
52	A_51_P417701 - Apaf1 (apoptotic peptidase activating factor 1)	0.219065166	0.25337386	0.381735245	0.367466108	1.156614101	1.038831165
53	A_51_P247799 - Casp8 (caspase 8, apoptosis-related cysteine peptidase)	1.550982599	0.489449731	0.937747479	1.128345305	0.315573967	0.831082006
54	A_52_P117576 - Casp3 (caspase 3, apoptosis-related cysteine peptidase)	0.113614502	0.246418709	0.100339392	0.113127455	2.168901898	0.886958802
55	A_55_P1968743 - Nfkb1(nuclear factor of kappa light polypeptide gene enhancer in B cells 1, p1(	0.52713219	1.365273642	0.456943185	0.472502249	2.590002409	0.967070921
56	A_55_P1968738 - Nfkb1(nuclear factor of kappa light polypeptide gene enhancer in B cells 1, p1(	0.651195502	1.551116944	0.572660182	0.589424159	2.381952792	0.971558721
57	A_51_P438666 - Keap1(kelch-like ECH-associated protein 1)	3.427946753	3.463824895	3.703179291	3.79786599	1.010466365	0.975068447
58	A_51_P411271 - Nfe2l2 (nuclear factor, erythroid derived 2, like 2)	18.28175922	34.14468218	18.46370278	18.396176	1.867691275	1.003670697
59	A_55_P2165869 - Cebpb (CCAAT/enhancer binding protein (C/EBP), beta)	28.96361352	51.97071492	34.99062915	34.85053746	1.794344994	1.004019786
60	A_52_P608322 - Maff (v-maf musculoaponeurotic fibrosarcoma oncogene family, protein F)	1.755270284	4.30104075	1.92542523	2.10585018	2.450358095	0.914322039
61	A_55_P2177023 - Mafg (v-maf musculoaponeurotic fibrosarcoma oncogene family, protein G)	4.859170988	15.5205739	5.035077554	5.001992866	3.194078566	1.006614301
62	A_55_P2177026 - Mafg (v-maf musculoaponeurotic fibrosarcoma oncogene family, protein G)	3.258451226	10.89146789	3.525952892	3.377794595	3.342529053	1.043862435
63	A_66_P116632 - Mafg (v-maf musculoaponeurotic fibrosarcoma oncogene family, protein G)	20.95441006	64.92384777	22.22348705	22.5518878	3.098338134	0.985437993
64	A_55_P2047748 - Mafg (v-maf musculoaponeurotic fibrosarcoma oncogene family, protein G)	6.302027282	23.32222457	7.540292035	7.444378968	3.700749541	1.012883958
65	A_55_P1953241 - Mafk (v-maf musculoaponeurotic fibrosarcoma oncogene family, protein K)	1.008209214	3.399426083	1.05495526	1.068461056	3.371746693	0.98735958
66	A_55_P2170454 - Gsta2 (glutathione S-transferase alpha 2)	0.009319264	0.115060536	0.007052737	0.009916763	12.34652608	0.711193461
67	A_51_P424338 - Nqo1(NAD(P)H dehydrogenase, quinone 1)	0.402763123	0.443422721	0.412452132	0.196859167	1.100951641	2.095163453
68	A_51_P365019 - Gclc (glutamate-cysteine ligase, catalytic subunit)	2.932332497	10.82289237	2.801351661	2.556717746	3.690881706	1.095682801
69	A_51_P320089 - Gclm (glutamate-cysteine ligase, modifier subunit)	0.563401199	1.803172352	0.613133187	0.592478969	3.200512095	1.034860677
70	A_55_P2029687 - Hmox1 (heme oxygenase (decycling) 1)	0.391591377	2.936790189	0.364830108	0.406271396	7.499629362	0.897996048
71	A_51_P263965 - Hmox1 (heme oxygenase (decycling) 1)	3.996637986	39.04096207	3.84818858	3.640141671	9.768450935	1.05715352
72	A_55_P2029574 - Por (P450 (cytochrome) oxidoreductase)	10.97784523	24.26837493	10.05080395	10.91844956	2.210668344	0.920533991
						Red; ≧1.5<br>Blue; ≦0.75	Red; ≧1.5<br>Blue; ≦0.75
